# An economic analysis of poliovirus risk management policy options for 2013–2052

**DOI:** 10.1186/s12879-015-1112-8

**Published:** 2015-09-24

**Authors:** Radboud J. Duintjer Tebbens, Mark A. Pallansch, Stephen L. Cochi, Steven G.F. Wassilak, Kimberly M. Thompson

**Affiliations:** Kid Risk, Inc., 10524 Moss Park Rd., Ste. 204-364, Orlando, FL 32832 USA; Division of Viral Diseases, National Center for Immunization and Respiratory Diseases, Centers for Disease Control and Prevention, Atlanta, GA USA; Global Immunization Division, Center for Global Health, Centers for Disease Control and Prevention, Atlanta, GA USA

## Abstract

**Background:**

The Global Polio Eradication Initiative plans for coordinated cessation of oral poliovirus vaccine (OPV) after interrupting all wild poliovirus (WPV) transmission, but many questions remain related to long-term poliovirus risk management policies.

**Methods:**

We used an integrated dynamic poliovirus transmission and stochastic risk model to simulate possible futures and estimate the health and economic outcomes of maintaining the 2013 *status quo* of continued OPV use in most developing countries compared with OPV cessation policies with various assumptions about global inactivated poliovirus vaccine (IPV) adoption.

**Results:**

Continued OPV use after global WPV eradication leads to continued high costs and/or high cases. Global OPV cessation comes with a high probability of at least one outbreak, which aggressive outbreak response can successfully control in most instances. A low but non-zero probability exists of uncontrolled outbreaks following a poliovirus reintroduction long after OPV cessation in a population in which IPV-alone cannot prevent poliovirus transmission. We estimate global incremental net benefits during 2013–2052 of approximately $16 billion (US$2013) for OPV cessation with at least one IPV routine immunization dose in all countries until 2024 compared to continued OPV use, although significant uncertainty remains associated with the frequency of exportations between populations and the implementation of long term risk management policies.

**Conclusions:**

Global OPV cessation offers the possibility of large future health and economic benefits compared to continued OPV use. Long-term poliovirus risk management interventions matter (e.g., IPV use duration, outbreak response, containment, continued surveillance, stockpile size and contents, vaccine production site requirements, potential antiviral drugs, and potential safer vaccines) and require careful consideration. Risk management activities can help to ensure a low risk of uncontrolled outbreaks and preserve or further increase the positive net benefits of OPV cessation. Important uncertainties will require more research, including characterizing immunodeficient long-term poliovirus excretor risks, containment risks, and the kinetics of outbreaks and response in an unprecedented world without widespread live poliovirus exposure.

**Electronic supplementary material:**

The online version of this article (doi:10.1186/s12879-015-1112-8) contains supplementary material, which is available to authorized users.

## Background

Since its launch in 1988, the Global Polio Eradication Initiative (GPEI) spearheaded interruption of indigenous wild poliovirus transmission (WPV) of all 3 serotypes in all but 3 countries (Afghanistan, Pakistan, Nigeria) by 2013 [1]. Since 2013, only serotype 1 WPV (WPV1) transmission has led to any laboratory-confirmed paralytic cases, with no detected indigenous serotype 2 WPV (WPV2) cases since 1999 [2] and no detected serotype 3 WPV (WPV3) cases since 2012 [3]. However, as long as any WPVs circulate anywhere, they can cause outbreaks in previously polio-free areas that do not maintain high population immunity through intense vaccination [4–6]. This provides further imperative to interrupt global WPV transmission as soon as possible. The live, attenuated oral poliovirus vaccine (OPV) remains the polio vaccine of choice in most countries because of its low costs, ease of administration, and proven ability to interrupt transmission in poor-hygiene settings by inducing good intestinal immunity and secondarily immunizing close contacts of OPV recipients [7]. However, OPV causes very rare vaccine-associated paralytic poliomyelitis (VAPP) in recipients and close contacts [7, 8]. Thus, ending all paralytic poliomyelitis disease (i.e., polio) requires global interruption of all WPV transmission and subsequent global cessation of OPV use [9]. In addition to relatively predictable VAPP cases that will stop as soon as OPV use stops, in populations with low immunity to poliovirus transmission, OPV-related viruses can continue to circulate and evolve to eventually acquire similar properties as WPVs, establish widespread transmission, and cause outbreaks of circulating vaccine-derived poliovirus (cVDPV) [8, 10–14]. The potential for cVDPVs motivates the requirement that countries globally coordinate OPV cessation and necessitates efforts to prepare for cVDPV outbreaks immediately after OPV cessation through intense surveillance, development of an outbreak response strategy, and creation of a global OPV stockpile for outbreak response [9, 15]. Moreover, long-term risks of vaccine-derived poliovirus (VDPV) reintroductions from rare chronic excretors with B-cell-related primary immunodeficiencies (i.e., iVDPVs) or intentional or unintentional release of any live poliovirus (LPV, i.e., WPV, VDPV, OPV, or OPV-related poliovirus) imply the need for continued management to ensure containment even after successfully-coordinated OPV cessation [8].

Most high-income countries use the injectable, inactivated poliovirus vaccine (IPV) exclusively for routine immunization (RI), and middle-income countries continue to adopt IPV for RI using a sequential schedule of IPV followed by OPV (IPV/OPV) or using an IPV dose co-administered with the third non-birth OPV dose [16, 17]. IPV remains much more expensive than OPV, but does not come with VAPP or cVDPV risks because it does not contain a LPV [18]. In anticipation of OPV cessation, the GPEI recommends and supports the addition of one IPV dose co-administered with the third OPV RI dose, which will provide some immunity for recipients to the serotypes stopped [19]. IPV generally provides better seroconversion (i.e., “take”) per dose for all three serotypes than OPV and it protects vaccinated individuals from polio [4]. However, IPV does not protect as well as OPV from infections or from participation in asymptomatic fecal-oral poliovirus transmission, and IPV remains untested in its ability to stop or prevent poliovirus transmission in developing countries [18, 20, 21]. Consistent with data from clinical trials showing limited intestinal immunity provided by IPV [20], despite IPV-only RI coverage over 90 %, Israel recently detected intense asymptomatic WPV1 transmission for 12 months likely due to relatively lower hygienic conditions in the Bedouin populations in the South [22, 23]. In the context of OPV cessation, we previously showed that adding IPV to RI provides only a very limited (if any) reduction of cVDPV risks after OPV cessation, because the populations most likely to experience cVDPV outbreaks are characterized by low RI coverage and intense, mostly fecal-oral transmission [24]. However, IPV may offer a relatively greater reduction in long-term global risks associated with iVDPV introductions or other releases and may help prevent sustained transmission of OPV-related viruses and thus cVDPV emergence in settings with higher RI coverage and less fecal-oral transmission [25]. As we move into the OPV cessation transition period, uncertainty remains and discussions will continue about the role of IPV.

A 2008 integrated economic analysis of global poliovirus risk management policies after the certification of global WPV eradication began with an assumption of cessation of all OPV use in 2010 to explore post-eradication immunization options [26, 27]. The analysis assumed that any outbreaks occurring after OPV cessation would remain contained within their populations of origin, which varied randomly in size between 500,000 and 100 million people [26, 27]. The analysis noted the need for further work to better characterize the spread between populations of both the outbreak virus and any OPV-related viruses used to respond to the outbreak [26, 27]. At the time of that analysis, we anticipated that low- and middle-income countries would continue to use trivalent OPV (tOPV) until coordinated tOPV cessation following the assurance of global interruption of all WPVs. The poliovirus transmission model used for the analysis [28] assumed continued intense tOPV use to maintain relatively high population immunity against all serotypes at the time of tOPV cessation, which supported a focus on the “average” serotype rather than each serotype. However, the GPEI subsequently shifted its focus to first interrupting WPV1 transmission using serotype 1 monovalent OPV (mOPV1) [29]. This led to gaps in population immunity to serotype 3 (and serotype 2), with ongoing WPV3 circulation necessitating the use of serotype 3 monovalent OPV (mOPV3) to respond to a resurgence of WPV3 cases. The licensure of bivalent serotype 1 and 3 OPV (bOPV) in 2010 allowed the use of bOPV to cover both circulating WPV serotypes. However, the decreased use of serotype 2-containing OPV (currently only available in tOPV) reduced population immunity to serotype 2 transmission and led to numerous serotype 2 cVDPV (cVDPV2) emergences, including several large and prolonged outbreaks [10, 13]. In part due to these outbreaks, the GPEI Strategic Plan 2013–2018 proposed phased globally-coordinated withdrawal of the OPV serotypes, starting with globally-coordinated cessation of serotype 2-containing OPV (i.e., OPV2 cessation, planned for April 2016) [30], regardless of the interruption of WPV1 and WPV3 [19]. The plan includes the introduction of at least one IPV dose into the RI schedules in OPV-using countries prior to OPV2 cessation [19]. While the plan anticipates simultaneous globally-coordinated cessation of serotype 1-containing OPV (OPV1) and serotype 3-containing OPV (OPV3) after 2018 (OPV13 cessation), the possibility of certification of global WPV3 interruption in 2016, while WPV1 may continue to circulate, raises the potential for phased withdrawal of OPV3 and then OPV1 [31]. Potential delays associated with stopping cVDPV2 circulation early enough to meet the April 2016 OPV2 cessation timing may also necessitate delay in OPV2 cessation, which could then motivate discussions about simultaneous withdrawal of OPV2 and OPV3 [32].

The use of serotype-specific OPV and increased adoption of IPV significantly changed both the space of endgame policy options [31] and the global starting conditions. Moreover, intense research and development efforts may change IPV costs, and recent evidence provides new data to inform cVDPV and iVDPV risks and to better characterize immunity to poliovirus transmission using models. Motivated by the evolving evidence, policy landscape, and population immunity levels across the world, this study presents an expanded integrated global model to assess the economics of polio endgame policies starting from 2013. No prior analysis considers global policies to achieve global WPV interruption and manage the endgame starting with the current GPEI strategic plan [19].

## Methods

### Analytical framework

We develop a model to characterize prospectively the economic outcomes associated with long-term poliovirus risk management policy options. The 40-year analytical time horizon runs from the beginning of 2013 (T_0_) through the end of 2052 (T_end_). The model considers 200 countries included in both the United Nations World Population Prospects (2012 revision) [33] and the World Bank list of economies (as of 2013) [34]. We categorize countries as low-income (LOW), lower middle-income (LMI), upper middle-income (UMI), and high-income (HIGH) according to the 2013 World Bank levels [34] to approximate the variability in conditions throughout the world. The analysis takes a global, societal perspective and thus includes all costs and benefits regardless of who pays or receives them. We use a 3 % discount rate [35, 36] for future costs and polio cases to report 2013 net present values and we use the United States Consumer Price Index [37] to convert all financial estimates to 2013 United States dollars ($), unless indicated otherwise.

Table 1 lists the policy options we consider, including two reference cases (RCs) that continue the *status quo* indefinitely with or without continued supplemental immunization activities (SIAs) (i.e., RC with SIAs, RC without SIAs). In all of our analyses, we assume that countries using an IPV-only or IPV/OPV sequential RI schedule at T_0_ will continue to use IPV for the entire analytical time horizon. However, for countries that use OPV at T_0_ we consider the current strategic plan through 2018 [19] followed by global minimum policies of IPV use for 5 or 10 years following OPV13 cessation (i.e., IPV5, IPV10), and IPV use through the end of the analytical time horizon (IPV through T_end_). We also consider a policy that follows the current plan for OPV2 cessation in 2016 and OPV13 cessation in 2019 except that countries using OPV-only at T_0_ do not introduce IPV (i.e., No IPV). We assume that these global IPV options reflect minimum requirements, with the expectation that countries can always opt to do more than the minimum recommended policy [31]. The model assumes that LOW and LMI countries that currently use OPV-only would opt for the minimum policies, but UMI countries that use OPV-only or IPV/OPV at T_0_ will use IPV through *T*_*end*_ regardless of the global minimum policies (Table 1).

**Table 1:** Main minimum global policy options considered for the economic analysis. We assume that countries using IPV-only at T_0_ will continue to do so indefinitely regardless of the policy choice.

Policy abbreviation	Description	Characterization in countries using OPV-only at T_0_ (LOW, LMI, or UMI)	Characterization in countries using IPV/OPV at T_0_ (UMI or HIGH)
RC with SIAs	Continued *status quo*	Continue tOPV-only indefinitely for RI supplemented with bOPV and tOPV SIAs	Continue IPV/OPV indefinitely supplemented with bOPV and tOPV SIAs
RC no SIAs	Continued *status quo*, but with no SIAs from 1/1/2019	Continue tOPV-only indefinitely for RI supplemented with bOPV and tOPV SIAs until 1/1/2019	Continue IPV/OPV indefinitely supplemented with bOPV and tOPV SIAs until 1/1/2019
IPV5	Current plan [19] with IPV everywhere for 5 years after all-OPV cessation	• Add IPV doses to RI schedule on 1/1/2015^a^	• Replace all tOPV with bOPV on 4/1/2016
		• Replace all tOPV with bOPV on 4/1/2016	• OPV13 cessation on 4/1/2019, switch RI to IPV-only indefinitely
		• OPV13 cessation on 4/1/2019^b^	
		• IPV cessation on 4/1/2024 in LOW and LMI countries	
IPV10	Current plan [19] with IPV everywhere for 10 years after all-OPV cessation	• Same as above but with IPV cessation of 4/1/2029 in LOW and LMI countries	• Same as above
IPV through T_end_	Current plan [19] with IPV everywhere until at least *T*_*end*_	• Same as above but without IPV cessation anywhere	• Same as above
No IPV	Current plan [19] but without global IPV use after OPV cessation of any type	• Replace all tOPV with bOPV on 4/1/2016	• Replace all tOPV with bOPV on 4/1/2016
		• OPV13 cessation on 4/1/2019	• OPV13 cessation on 4/1/2019, switch RI to IPV-only indefinitely

*Abbreviations:* bOPV, bivalent OPV (serotypes 1 and 3); HIGH, high-income; IPV, inactivated poliovirus vaccine; LMI, lower middle-income; LOW, low-income; OPV, oral poliovirus vaccine; OPV13 cessation, globally-coordinated cessation of OPV containing serotypes 1 and 3; RC, reference case; RI, routine immunization; SIA, supplemental immunization activity; T_0_, beginning of analytical time horizon (i.e., January 1, 2013); T_end_, end of analytical time horizon (i.e., December 31, 2052); tOPV, trivalent OPV; UMI, upper middle-income

^a^ Assumes a single IPV dose administered synchronously with OPV to any child that receives at least one non-birth OPV RI dose in LOW and LMI countries, but a sequential IPV/IPV/OPV/OPV schedule in UMI countries

^b^ Assumes LOW and LMI countries continue with a single-dose IPV schedule while UMI countries switch to a 3-dose IPV-only RI schedule indefinitely

We compute the incremental cost-effectiveness ratio (ICER) and incremental net benefits (INB) of each alternative option compared to each RC, which continues the *status quo* indefinitely and encompasses the spectrum of SIA frequencies that might occur with continued OPV use. We express the ICER in $ per prevented (paralytic) polio case and $ per disability-adjusted life-year (DALY) [38] averted and the INB in $ (see equations in Additional file 1). Negative values for ICERs distort the interpretation of these ratios [27, 39]. For example, an intervention with $100 in incremental costs but one more case compared to the *status quo* represents an undesirable option but receives the same ICER of −100 $/case as a desirable intervention that saves $100 and prevents one case. Therefore, we refer to ICERs with negative incremental costs and negative prevented cases as *cost-saving but life-costing* (CSLC), those with negative incremental costs but positive prevented cases as *cost- and life-saving* (CLS), and those with positive incremental costs but negative prevented cases as *dominated* [26]. Given complications associated with aggregating ICERs across different income levels, we report the ICER separately for each income level, while we report the INB both by income level and as a global aggregate [26, 40].

We implemented the model in JAVA^TM^ using Eclipse^TM^ and perform model runs on the Amazon Elastic Compute Cloud. We perform 100 stochastic iterations of the model for all considered policy options, and we use the results to characterize the economic metrics based on the average annual costs and cases by income level. For the RCs, one iteration suffices, because they do not include the stochastic poliovirus reintroduction events after OPV cessation, and we confirmed limited impact of random poliovirus exportations in the context of continued OPV use.

### Economic inputs

Table 2 shows the economic model inputs. The GPEI Financial Resource Requirements (FRRs) [41] of the current six-year plan for 2013–2018 [19] include a total of $4.1 billion in resources budgeted to individual countries, and another $1.4 billion of global programmatic costs for technical assistance, surveillance and the Global Polio Laboratory Network, and other global-level and regional-level costs not budgeted to any individual countries. We assume that all policy options would incur approximately the same global programmatic costs, and therefore we do not include these in the incremental results. We calculate immunization costs associated with delivering vaccine doses based on unit cost estimates from prior work [18, 40, 42]. We attribute any difference between our estimates of aggregate costs based on this approach and those budgeted in the FRRs to costs not covered by GPEI resources, such as national resources for RI (including in-kind contributions), volunteer time [43], and any bilateral funds not included in the FRRs.

**Table 2:** Economic model inputs by World Bank income level [34] for vaccine, treatment, and societal costs in 2013 United States dollars ($), with earlier estimates converted using the United States Consumer Price Index. [37]

Model input	Base Case Value				Source
	Low-income country	Lower middle-income country	Upper middle-income country	High-income country	
Vaccine price per dose					[18,73]
- OPV (any formulation)	$ 0.12	$ 0.12	$ 0.13	$ 0.16	
- IPV (10-dose vial)	$ 1.30	$ 2.30	$ 3.20	$ 13.00	
Effective vaccine wastage					[18,42,44]
- OPV in RI	50 %	50 %	30 %	10 %	
- OPV or IPV in SIAs	44 %	44 %	44 %	44 %	
- IPV (10-dose vial)	40%^a^	40%^a^	30 % or 25%^a^	10 % or 5%^a^	
Administration costs per dose					[18,42]
- OPV in RI	$ 0.86	$ 0.86	$ 2.29	$ 2.90	
- OPV co-administered in RI^b^	$ 0.30	$ 0.30	N/A	N/A	
- OPV in pSIAs	$ 0.60	$ 0.60	$ 3.30	$ 4.20	
- OPV in oSIAs^c^	$ 0.90	$ 0.90	$ 4.95	$ 6.30	
- IPV single antigen in RI	$ 1.08	$ 1.08	$ 2.86	$ 10.36	
- IPV combo in RI	N/A	N/A	$ 0.72	$ 2.59	
Treatment costs per paralytic polio case	$ 650	$ 6,500	$ 65,000	$ 650,000	[26,40]
Disability-adjusted life-years per paralytic polio case	13	14	14	14	[26,40]
Societal economic costs per paralytic polio case^d^	$ 7,800	$ 27,000	$ 96,000	$ 550,000	[40,46]

*Abbreviations:* IPV, inactivated poliovirus vaccine; N/A, not applicable; OPV, oral poliovirus vaccine; oSIA, outbreak response SIA; pSIA, planned, preventive SIA; RI, routine immunization; SIA, supplemental immunization activity

^a ^ Based on estimates for single IPV-dose in low and lower middle-income countries, and 2 (sequential) or 3 or more IPV doses (IPV-only) in the RI schedule, respectively, with lower values than prior estimates [18] to reflect the subsequently modified WHO open vial policy [74]

^b^ Incremental cost of for OPV co-administered with an IPV dose; estimate based on judgment

^c ^ oSIA administration costs assume 1.5 times the costs for pSIAs [42]

^d^ Based on DALY estimate, multiplied by the average annual per-capita gross national income of $597 for 33 low-income countries, $1,898 for 45 lower middle-income countries, $6,885 for 45 upper middle-income countries, and $39,091 for 49 high-income countries [46]. No estimates were available for 3 low-income countries (i.e., Democratic Republic of Korea, Myanmar, and Somalia), 3 lower middle-income countries (i.e., Djibouti, West Bank and Gaza, Syrian Arab Republic), 5 upper middle-income countries (i.e., Argentina, Belize, Cuba, Iran, Libya), and 17 mostly small high-income countries (i.e., Bahrain, Brunei Darussalam, Israel, Kuwait, New Zealand, Oman, Qatar, Saudi Arabia, and small island nations or extra-territorial states with autonomous status) otherwise included in the analysis.

We base our SIA cost estimates directly on the unit cost inputs in Table 2 and the targeted numbers of children vaccinated during SIAs estimated by the model and adjusted for wastage [44]. Table 2 assumes that the administration costs per OPV dose during outbreak response SIAs (oSIAs) equal 1.5 times those during regular, planned preventive SIAs (pSIAs) [42]. In addition to treatment costs, the INB accounts for the societal costs associated with lost wages and suffering due to polio. In the absence of any direct estimates, we approximate these by equating each DALY associated with polio with the appropriate average annual per capita gross national income [26, 27, 36, 40, 45, 46].

### Global poliovirus transmission model

We expanded and revised the poliovirus transmission model [28] used in the 2008 economic analysis [26, 27], to address the more complex policy space [47]. Specifically, the differential equation-based expanded poliovirus transmission and OPV evolution model (i.e., the DEB model): (1) characterizes each serotype separately (to analyze serotype-specific vaccination policies and risks), (2) considers explicitly both fecal-oral and oropharyngeal transmission (to account for the differential impact of IPV on fecal and oropharyngeal excretion), (3) uses 8 recent immunity states to reflect immunity derived from maternal antibodies, only IPV vaccination, only LPV infection, or both IPV vaccination and LPV infection (to more realistically capture the differences in immunity derived from IPV and LPV), (4) includes multi-stage waning and infection processes (for more realistic characterization of these processes), (5) characterizes OPV evolution as a 20-stage process from OPV as administered to fully-reverted polioviruses with assumed identical properties to typical homotypic WPVs (to allow cVDPV emergence to occur within the model), and (6) accounts for heterogeneous preferential mixing between mixing age groups and subpopulations [47] (Additional file 1).

For this analysis, we adopt all generic model inputs from the DEB model [47, 48]. We further scale the model up to a global level by characterizing global variability and mixing between populations. In the context of limited information to characterize all countries and heterogeneity within them and finite computing resources, we developed a simplified global model that does not explicitly identify individual countries or populations. Instead, it stratifies the world into 71 epidemiological blocks that each consist of 10 subpopulations with approximately 10 million people at T_0_ (i.e., a global population of 7.1 billion people). A subpopulation corresponds to a population with spatially homogeneous but age-heterogeneous mixing such as a country, state, or large city within a large country, or a group of well-connected smaller countries of the same income level. A block corresponds to a larger epidemiological area such as parts of very large countries (e.g., Western Uttar Pradesh in India, the northern states of Nigeria), large countries (e.g., Egypt, Ethiopia, Philippines), or groups of connected countries (e.g., Central Africa, West Africa minus Nigeria). Table 3 provides a breakdown of the global population as of 2013 [33] by World Bank income level [34] and polio vaccine use as of October 2012 [16] for all 200 countries with available data. Table 3 also allocates the 71 blocks to the different combinations of income level and polio vaccine use at T_0_. For this allocation, in some cases we assigned countries smaller than a block to a block with a higher or lower income level because of geographic proximity to countries that used the same polio vaccine in 2013 that probably incurred similar vaccine expenditures and transmission conditions.

**Table 3:** Distribution of the global population as of 2013 [33] in hundreds of millions by World Bank income level [34] and polio vaccine use as of October 2012 [16] covering 200 countries with available data (i.e., 99.7 % of the global population) with numbers in parentheses indicating the number of corresponding epidemiological blocks in the global model

Income level	Polio vaccine use at T_0_				*Total blocks*
	Unknown	OPV-only	IPV/OPV	IPV-only	
Unknown	0.233 (0)	0 (0)	0	0	0
LOW	0 (0)	8.46 (8)	0	0	9
LMI	0 (0)	24.2 (25)	0.67 (0)	0	25
UMI	0 (0)	18.7 (19)	5.31 (7)	0.39 (0)	25
HIGH	0 (0)	0.28 (0)	2.32 (2)	10.21 (10)	12
*Total blocks*	*0*	*52*	*9*	*10*	*71*

*Abbreviations:* HIGH, high-income; IPV, inactivated poliovirus vaccine; LMI, lower middle-income; LOW, low-income; OPV, oral poliovirus vaccine; T_0_, beginning of analytical time horizon (i.e., January 1, 2013); UMI, upper middle-income

We characterize random periodic infective interactions with people from other subpopulations and blocks. Specifically, we track the cumulative number of effective infections (CEI, defined as the cumulative prevalence of infectiousness-weighted infectious people) in each subpopulation, by virus reversion stage. Whenever the CEI of a reversion stage reaches a certain exportation threshold (E*) we trigger a potentially effective introduction of virus from the same reversion stage into another subpopulation and reset the CEI to zero. The exportation of poliovirus may or may not lead to an effective introduction that establishes transmission depending on micro-level dynamics not explicitly captured in the DEB model, and chance (e.g., the precise location of the virus introduction that impacts whether the infection spreads beyond the first household(s)) [49]. Therefore, we randomly determine if the exportation leads to an effective introduction, defined as an introduction that establishes subpopulation-wide transmission (i.e., transmission beyond the individual(s) importing the virus and its immediate surrounding household or community), using a function for the probability of an effective introduction (PEF), which logically depends on the immunity level of the receiving subpopulation. We model the PEF as a function of the mixing-adjusted net reproduction number (R_n_), which represents the average number of secondary infections generated by a single infection accounting for population immunity calculated as the basic reproduction number (R_0_) multiplied by the effective susceptible proportion [50]. R_n_ in a subpopulation depends on the baseline R_0_ of the subpopulation and the virus strain (i.e., different R_0_ values for different serotypes and reversion stages) [47, 48] and changes with time depending on vaccination policies, any immunity derived from LPV exposure, and seasonality. Thus, PEF depends on all of these factors through R_n_ (Additional file 1).

Effective virus introductions may or may not lead to an outbreak (i.e., at least one polio case) depending on the population immunity level in the receiving subpopulation and the kinetics of the initial infections relative to the seasonally changing R_0_. However, if they do, an outbreak can unfold very quickly in the model due to the assumption of homogeneous mixing within relatively large subpopulations of approximately 10 million people. The homogenous mixing assumption implies faster propagation of the virus than would occur if in reality the subpopulation remains more heterogeneous [47, 51]. Given the inability to observe ineffective introductions that die out locally due to chance or locally effective introductions that do not continue to circulate due to high surrounding population immunity, and the reality of spatial heterogeneity in mixing within subpopulations, estimating the exportation threshold E* from data on long-range exportations remains challenging.

Based on the relatively localized transmission of cVDPVs to date despite presumably large numbers of infections (e.g., Nigeria) [13], we determine E* such that a cVDPV2 outbreak in a subpopulation within a year following OPV2 cessation yields approximately one expected effective exportation to another subpopulation, assuming an aggressive and effective response in the subpopulation of the initial outbreak. This criterion leads to an estimate of E* of 200,000 CEIs and remains consistent with our current experience associated with cVDPV and WPV importation outbreaks in the context of populations with recent widespread LPV exposure. We remain uncertain about the kinetics of poliovirus transmission between populations in the unprecedented context of no recent global LPV exposure as the time since OPV cessation increases, but our model assumes that the inherent frequency of potentially effective exportations per CEI (i.e., E*) does not change over time. However, the probability that an exportation becomes effective in the receiving subpopulation increases in the absence of recent LPV exposure through the dependence of PEF on R_n_, which increases rapidly after OPV cessation in high-R_0_ populations, regardless of IPV use [24, 52]. Similarly, the outbreak kinetics following an effective introduction speed up as the time since OPV cessation increases.

Assuming preferential mixing between the subpopulations in an epidemiological block, we assume that 24 out of 25 (96 %) exportations go to random subpopulations within the same block, while the remaining 1 out of 25 (4 %) go to random subpopulations of other blocks (i.e., inter-block exportations). Thus, inter-block exportations occur once every 5 million CEIs on average (i.e., 1/200,000 × 1/25). To determine the importing block for an inter-block exportation, we group all 71 blocks into 9 regions with variable number of blocks, corresponding to large geographical regions (i.e., Africa, Australasia, China and neighbors, East and Central Asia, Europe, India, Latin America and the Caribbean, North America, and South Asia) (Additional file 1). We assume that 3.5 % of all exportations (i.e., 87.5 % of all inter-block exportations) go to a random block in the same region and that the remaining 0.5 % (i.e., 12.5 % of all inter-block exportations) go to a random block in a different region. To characterize the global variability in conditions relevant to poliovirus transmission, we vary a number of model inputs by subpopulation related to viral transmission (i.e., R_0_ and its seasonal fluctuations, the relative importance of oropharyngeal and fecal-oral poliovirus transmission) and immunization program performance (i.e., OPV take rates, RI and SIA intensity, and surveillance quality)(Additional file 1). To approximate the WPV prevalence and global immunity levels at T_0_ we run the model for a “burn in” period to begin the policy comparisons starting with initial conditions that approximate actual demographic profiles and exposure histories [47] (Additional file 1).

### Simulation of post-OPV cessation risks

We assume tOPV intensification leading up to OPV2 cessation maximizes population immunity at OPV2 cessation and avoids cVDPV2 emergences after OPV2 cessation [24, 52] and sufficient bOPV use before OPV13 cessation to avoid subsequent cVDPVs. Thus, we focus on other risks, including the small, but non-zero probabilities of unintentional or intentional release of LPV and introduction of iVDPVs from prolonged or chronic excretors [8, 53, 54]. Table 4 provides estimates for the non-cVDPV risks based on the currently available evidence [54] and updated from prior work [8].

**Table 4:** Global model inputs that do not vary between blocks, characterization of oSIAs, and characterization of non-cVDPV risks and potential polio antiviral drug use

Model input	Value
Age groups	0-2, 3–11 months; 1–4, 5–9, 10–14, 15-39^a^; ≥ 40 years^a^
Number of equally-sized subpopulations per block	10
Proportion of children receiving fewer than 3 non-birth RI doses who receive 1 non-birth dose	0.2
Proportion of children receiving fewer than 3 non-birth RI doses who receive 2 non-birth doses	0.2
Relative coverage with birth dose compared to non-birth RI coverage with 3 doses	
- LOW, LMI blocks that use OPV-only at T_0_	0.5
- All other blocks	0
Average per-dose take rate for IPV	
- LOW, LMI	0.63
- UMI	0.70
- HIGH	0.75
Duration of each SIA (days)	5
Number of oSIA rounds	
- Before homotypic OPV cessation	3
- After homotypic OPV cessation, R_0_ < 12	4
- After homotypic OPV cessation, R_0_ ≥ 12	6
Geographical scope of oSIAs	
- Before homotypic OPV cessation	Subpopulation
- After homotypic OPV cessation, R_0_ < 10	Subpopulation
- After homotypic OPV cessation, R_0_ ≥ 10	Block
Target age groups	Cohorts born since OPV cessation, rounded to next multiple of 5
oSIA impact	
- True coverage	0.8
- Repeated missed probability	0.7
Time from outbreak detection until the first oSIA (days)^b^	
- No ongoing outbreak response in block	45
- Outbreak response already ongoing in block	30
Interval between oSIA rounds (days)	30
Number of years when mOPV allowed for oSIAs after OPV cessation of each type (years)	5
Exportation threshold (*E* ^*^, i.e., cumulative effective infections needed to trigger a potential exportation from a subpopulation)	200,000
Proportion of virus exportations	
- within the same block	0.960
- in another block within the same region	0.035
- outside of the region	0.005
Characterization of post-OPV cessation risks (non-cVDPV)	
Average time between contacts of long-term iVDPV excretors with the general population (days)	150-600
Global Poisson rate^c^ for release of unreturned OPV (only during first year after OPV cessation of each type and in blocks that use OPV at T_0_) (1/year)	0.1
Global Poisson rate^c^ for release from IPV production site (1/year)	0.2
Global Poisson rate^c^ for other unintentional or intentional release (1/year)	0.025
Probability that other unintentional or intentional release is unintentional	0.5
Distribution of unintentional releases by income level	
- LOW	0
- LMI	0.01
- UMI	0.09
- HIGH	0.90
Distribution of intentional releases by income level	
- LOW, LMI, UMI	0.5
- HIGH	0.5
Characterization of impacts of PAVDs	
Proportion of long-term iVDPV excretors who had VAPP that receive PAVDs	
- No PAVDs (base case)	0
- PAVD40%	0.5
- PAVD90%	0.9
Proportion of asymptomatic long-term iVDPV excretors that receive PAVDs	
- No PAVDs (base case)	0
- PAVD40%	0
- PAVD90%	0.9
Proportion of long-term iVDPV excretors receiving PAVDs who recover	
- PAVD40%	0.4
- PAVD90%	0.9

*Abbreviations:* cVDPV, circulating vaccine-derived poliovirus; HIGH, high-income; IPV, inactivated poliovirus vaccine; iVDPV, immunodeficiency-associated vaccine-derived poliovirus; LMI, lower middle-income; LOW, low-income country; OPV, oral poliovirus vaccine; oSIA, outbreak response SIA; PAVD(40 %, 90 %), polio antiviral drug (passive or active use policy, respectively); R_0_, basic reproduction number for serotype 1 wild poliovirus; RI, routine immunization; T_0_, beginning of analytical time horizon (i.e., January 1, 2013); SIA, supplemental immunization activity; UMI, upper middle-income

^a^ Age groups impacting the fraction of newborns born as maternally immune children [47,52]

^b^ Detection of paralytic cases assumes a time of 10 days between onset of infection and paralysis to reflect the average incubation period [47]

^c^ Global Poisson rates indicate the baseline annual rate at which potential introduction events occur anywhere in the world, with the distribution by income level indicated separately or as indicated in the text for IPV production site releases

For the iVDPV risks, we constructed a discrete-event simulation (DES) model of long-term iVDPV excretor prevalence to estimate iVDPV prevalence until and after OPV cessation of each serotype [54]. For each stochastic iteration of the global model, we use one stochastic realization of the DES model to generate random introductions of iVDPV into the general population after OPV cessation. We randomly generate contacts with the general population for each individual with active long-term iVDPV excretion after OPV cessation. To estimate the rate of general population contacts, we assume that R_0_ provides a measure of the average number of contacts per approximately 30 days for immunocompetent individuals, assuming approximately 30 days of excretion for fully susceptible individuals [47]. While we model R_0_ as ranging from 4–13 globally [47], we assume that any primary immunodeficiency disease (PID) patients surviving long enough to become long-term excretors in any setting mix much less intensely with others than immunocompetent individuals in the general population (i.e., their continued survival depends on relatively good hygiene and limited mixing), with their R_0_ values effectively ranging from 1–4. We further assume that the majority of contacts (i.e., 95 %) involve close contacts (e.g., in the same household) with individuals who possess sufficient immunity to prevent further spread due to their ongoing exposure to the long-term excretor. This leaves between 0.05 and 0.2 contacts (i.e., R_0_ of 1–4 times 5 % of contacts that are not close) per 30 days with the general population for a long-term excretor, or an average time of approximately 150–600 days between potential contacts that may lead to an iVDPV infection in the general population (Table 4). We draw a random contact rate for each individual long-term excretor from this range with a uniform distribution. Based on the contact rate for the individual long-term excretor, we randomly determine the time between general population contacts and include as potentially effective iVDPV introductions all contacts until (1) the excretor dies, (2) the excretor recovers and stops excreting, or (3) the time of the next contact exceeds the analytical time horizon (i.e., it would occur after 2052). In addition to using the DES model [54] to track the prevalence of and generate potentially effective introductions from long-term excretors infected prior to OPV cessation, we also use it to account for the possibility of creating new iVDPV excretors exposed to any mOPV used to respond to outbreaks after OPV cessation (Additional file 1). As with LPV importations, iVDPV excretor contacts with the general population or other releases of poliovirus may or may not lead to effective introductions depending on micro-level dynamics and chance, and therefore we apply the PEF to determine whether the introduction establishes transmission.

For IPV production sites releases, we assume that ongoing production of IPV from WPV seed strains will continue indefinitely in 5 fixed different subpopulations in HIGH blocks, which may generate potential WPV introductions at any time. We assume that a further 7 (for IPV5 or IPV10) or 10 (for IPV through T_end_) facilities in non-HIGH subpopulations from a selected list of blocks that use OPV-only at T_0_ will produce IPV from Sabin seed strains (Additional file 1). These sites may generate potential OPV introductions as long as IPV remains in use in the corresponding block, which depends on the policy option. The literature documents 4 reported containment failures during the past 25 years of IPV production [8, 55, 56], and one additional breach of containment from an OPV production site [8]. While improved containment guidelines may reduce this risk, some releases may have gone unnoticed due to currently very high population immunity to transmission, and global IPV production will increase. Therefore, we assume a continued rate of IPV production site releases of 1 per 5 years, independent of the number and locations of IPV production sites. Upon triggering a vaccine production site release, the model randomly selects one of the assumed production sites. The location determines the type of virus released (i.e., WPV from a current production site in a HIGH block or OPV from a non-HIGH Sabin-IPV production site), while the model randomly selects the serotype (each with equal probability). The selection of the virus determines its transmission properties, and we randomly determine the probability that the release comprises an effective introduction based the R_n_-dependent PEF, as for other releases or virus importations. Although potential future research may develop non-replicating IPV seed strains, we do not consider that possibility in this analysis.

We assume much lower rates for other releases, translating into an approximately 10 % chance of a release of unreturned OPV during the first year after OPV cessation, and 10 % chance of any other intentional or unintentional release at any point during the analytical time horizon (Table 4). If any of these releases occurs, we randomly select the OPV (i.e., in the event of an unreturned OPV release) or WPV serotype released with equal probability, and the receiving subpopulation according to the assumed distribution of the risk by income level in Table 4. To ensure comparability across policies, we use the same list of potential post-OPV-cessation introduction events for all policy options, and in some cases the policy choice affects whether the potential introduction takes place.

### Characterization of outbreak response after OPV cessation

Table 4 includes model inputs related to oSIAs. Once a block eliminates WPV, but before OPV cessation, we start accumulating the incidence of polio cases in each subpopulation resulting from effective importations or indigenous cVDPV emergences. If the cumulative incidence of WPV or fully-reverted VDPV cases per 10 million people reaches more than the subpopulation-specific detection threshold (i.e., 1, 2, or 3 polio cases), then this triggers outbreak response SIAs (oSIAs) in the subpopulation that override any scheduled pSIAs and that start at 45 days after detection. After the oSIAs, the subpopulation returns to its post-WPV-elimination schedule (Additional file 1) and again begins accumulating polio cases from WPV or fully-reverted VDPV until any new detection occurs. After global OPV cessation of a serotype, the nature of outbreak response changes. We accumulate polio cases from any LPV (i.e., all OPV-related viruses) to trigger oSIAs and we modify the response strategy to reflect sufficiently aggressive response to minimize the chances of failing to fully control outbreaks after OPV cessation, as shown in Table 4. In the event of a subpopulation-specific response after OPV cessation, we assume that all other subpopulations in the same block remain on “high alert” between detection and the completion of the last oSIA in the outbreak subpopulation, characterized as high acute flaccid paralysis (AFP) surveillance quality (i.e., detection after occurrence of 1 cumulative paralytic case per 10 million people) and a short response delay (i.e., 30 days between detection and the first oSIA).

We assume that subpopulations that use IPV-only at T_0_ would use only IPV for oSIAs any time after they switch to IPV-only and particularly after global OPV cessation, based on the unavailability of OPV for outbreak response in the United States (i.e., the largest IPV-only country to date) [57, 58], although some IPV-only countries responded to outbreaks with both IPV and OPV [22, 59]. For all other subpopulations, the vaccine choice depends on time and the detected serotype. Specifically, before OPV cessation of any serotype oSIAs use tOPV (if serotype 2 poliovirus detected) or bOPV (if no serotype 2 poliovirus detected), during the first 5 years after OPV cessation of any serotype they use mOPV of the detected serotype, and any time after that they use IPV, because we assume that the risk related to reintroducing large amounts of LPV becomes too large to use OPV that long after OPV cessation. The optimal duration of mOPV use for oSIAs after OPV cessation remains uncertain, but 5 years resulted in a very low probability (i.e., <1 %) of exported OPV-related viruses establishing transmission in other subpopulations or blocks, given all other model assumptions. We do not constrain the amount of mOPV and IPV available for oSIAs after OPV cessation, which allows us to estimate potential vaccine needs from the stockpile, based on the total targeted population in all oSIAs after OPV cessation, adjusted for the estimated wastage rates during SIAs (Table 2). We report the fraction of stochastic iterations in which for at least one serotype the number of mOPV doses needed for oSIAs exceeds the 500 million total and 100 million filled mOPV doses of each serotype currently planned for the stockpile.

### Variations of the IPV5 policy

In the context of the IPV5 policy, we consider the potential impact of the adoption of polio antiviral drugs (PAVDs) to treat iVDPV excretors from 2017 forward, which would potentially clear their infections [54]. We consider IPV5 with PAVD passive use as one option, which assumes 40 % effectiveness in clearing the infection with treatment of 50 % of excretors with paralysis on January 1, 2017 and of those who subsequently develop paralysis (i.e., IPV5, PAVD40%). We also consider IPV5 with PAVD active use, which assumes 90 % effectiveness in clearing the infection and treatment of 90 % of all excretors with an ongoing infection after January 1, 2017 (i.e., IPV5, PAVD90%). We randomly pre-determine which excretors would recover from their iVDPV infections as a result of PAVD treatment based on the probabilities for the two scenarios, which provide some bounds on the combined effectiveness of the PAVD compound(s) and the degree of passivity of efforts to identify and treat iVDPV excretors. We also consider the impact of a failure to intensify tOPV use leading up to OPV2 cessation, which assumes continuation of the SIA schedule from before the year 2015 up until the time of OPV2 cessation (Additional file 1). Finally, we consider the impact of a higher E* and lower cumulative paralytic case thresholds used to trigger an OPV restart (compared to the base case threshold of 50,000 cumulative polio cases after 2016 above which we assume countries that used OPV as of 2013 would restart using OPV).

## Results

### Expected future burden of polio cases

Table 5 reports the average total number of cases (including VAPP) over the analytical time horizon, broken down by iterations with or without OPV restart, the number of iterations with uncontrolled outbreaks leading to OPV restart in all countries that use OPV at T_0_ (i.e., the number of runs reaching 50,000 cumulative cases), and the number of iterations with any detected outbreaks that trigger a response. For all policies involving OPV cessation, ≥96 % of iterations involve one or more outbreaks after OPV cessation, which implies expected use of the vaccine stockpile and outbreak response plans. The majority of the outbreaks trace back to long-term iVDPV excretors, who can re-introduce polioviruses years after OPV cessation when population immunity to transmission becomes low enough to allow these viruses to establish transmission and cause outbreaks. However, the longest expected survival of iVDPV excretors occurs in lower-R_0_ settings with less fecal-oral transmission [54] in which IPV provides more impact on poliovirus transmission. In higher-R_0_ settings we expect few long-term iVDPV excretors to survive beyond the 5-year window during which our model allows mOPV use for oSIAs.

**Table 5:** Undiscounted, average total cases for the main minimum global policy options and number of iterations with OPV restart and with any outbreaks requiring a response for 100 stochastic iterations

Result	RC with SIAs	RC no SIAs	IPV5	IPV10	IPV through T_end_	No IPV
Average number of cases after type-specific OPV cessation, 2013-2052^a^	6,800	1,600,000				
- No OPV restart^b^			340	120	470	840
- OPV restart with SIAs			350,000	320,000	120,000	170,000
- OPV restart without SIAs			720,000	680,000	540,000	880,000
- All iterations, OPV restart with SIAs			7,300	6,400	12,000	11,000
- All iterations, OPV restart without SIAs			15,000	14,000	55,000	54,000
Number if iterations with OPV restart	N/A	N/A	2	2	10^c^	6
Number of iterations with one or more post-OPV cessation outbreak response	N/A	N/A	96	96	96	100

*Abbreviations* *(see Table * 1 *for policy abbreviations):* IPV, inactivated poliovirus vaccine; N/A, not applicable; OPV, oral poliovirus vaccine; SIA, supplemental immunization activity; T_end_, end of analytical time horizon (i.e., December 31, 2052)

^a ^Does not include a total of approximately 1,150 cases (i.e., approximately 1,100 VAPP, 80 WPV1, and 3 cVDPV2 cases) before OPV cessation of each type for the two reference cases or the No IPV options and approximately 1,000 cases (i.e., approximately 920 VAPP, 80 WPV1, and 3 cVDPV2 cases) before OPV cessation of each type for the policies that involve IPV use everywhere

^b^ OPV restart defined as the occurrence of at least 50,000 polio cases since 2016 and by 2051, leading to OPV restart at the beginning of 2052 or earlier

^c^ In addition, in 4 other iterations, the model included ongoing transmission of live poliovirus at the end of the analytical time horizon, but the cumulative number of cases did not hit the contingency of 50,000 yet

Aggressive outbreak response rapidly controls the majority of the expected outbreaks, avoiding exportations to other subpopulations and blocks that would lead to a high number of cases after OPV cessation and eventual OPV restart. However, for IPV5 and IPV10, 2 of 100 iterations led to poliovirus reintroductions that occurred at a time and place with very low IPV-only-induced population immunity (i.e., due to a combination of high enough R_0_ and contribution of fecal-oral transmission and introduction long enough after OPV cessation) that triggered an OPV restart. One of these traced back to an iVDPV1 introduction in a very high-R_0_ block relatively soon after OPV cessation, which triggered mOPV SIAs that infected a PID patient who became a new long-term excretor and reintroduced an iVDPV1 at a time when the model no longer allows mOPV use for outbreak response. The other traced back to an unintentional or intentional “other” release (i.e., from an accidental breach in laboratory containment or a bioterrorism event) of WPV3 in a LMI block with an R_0_ of 8 in the second half of the year 2049. As in most relatively higher R_0_ populations, we observed for the first OPV restart iteration that even a very large number of oSIAs with IPV with a block-wide geographical scope and increasingly wide target age range could not control the outbreak. While the IPV oSIAs kept the incidence relatively low and delayed spread to other blocks for many years, eventually enough effective exportations occurred to trigger new large outbreaks and accumulate over 50,000 cases. These two iterations provide two examples from a larger number of possible scenarios that could potentially lead to an OPV restart and they average between approximately 300,000-700,000 expected polio cases, depending on whether the OPV restart involves resumed SIAs. However, based on 100 stochastic iterations, any such scenario represents a relatively rare event in the context of our assumed aggressive outbreak response and frequency of spread between populations. The 98 iterations that control all outbreaks with IPV5 average an expected 340 post-OPV-cessation polio cases.

The policy of IPV through T_end_ led to a total of 10 OPV restarts, including the 2 that occurred with IPV5 or IPV10 and 8 additional iterations associated with release of Sabin seeds strains from Sabin IPV (sIPV) production sites after the year 2035 and located in blocks with a R_0_ between 9 and 11. This led us to the general observation that in some relatively high-R_0_ blocks (e.g., R_0_ ≥ 9), any releases of Sabin seed strains can eventually establish uncontrollable transmission. Thus, based on the historical rate of releases from poliovirus vaccine production sites during the last 25 years, the use of any LPV strains in high-R_0_ populations presents an important risk that requires management. Releases of WPV and Sabin seed strains also occurred in lower-R_0_ blocks in some iterations, but these either did not establish any transmission (e.g., Sabin seed strain releases) or led only to smaller, controlled outbreak in high-income blocks (WPV seed strain releases).

The policy of No IPV use assumes that all blocks that use OPV-only at T_0_ do not add IPV at any time before or after OPV cessation. The absence of any IPV use in these blocks allows population immunity to drop more rapidly, particularly in medium-R_0_ populations (i.e., between 6–8) in which we expect better survival of long-term iVDPV excretors. Consequently, for this policy option we observed 5 iterations in which iVDPV introductions triggered an eventual OPV restart (in addition to one OPV restart associated with an “other” release that caused an OPV restart for all policies), including one new iVDPV excretor associated with mOPV use for outbreak response after OPV cessation (Additional file 1).

For the global IPV policies (i.e., IPV5, IPV10, or IPV through T_end_), the estimated number of mOPV doses needed from a stockpile for use in oSIAs after OPV cessation exceeded the currently planned 100 million filled mOPV doses for at least one serotype in 32 stochastic iterations (i.e., including 23 iterations for mOPV1, 12 for mOPV2, 8 for mOPV3). Given that all mOPV use in the model occurs within 5 years of OPV cessation of each serotype, this suggests a high probability of needing to fill some of the bulk mOPV stock soon after OPV cessation. In 2 stochastic iterations, the estimated number of mOPV doses exceeded the currently planned total stockpile of 500 million mOPV doses for at least one serotype (i.e., 1 for mOPV1 and 1 for mOPV3). One of those 2 iterations led to an OPV restart even in the event of an unlimited stockpile. In the other iteration, exhaustion of the entire mOPV stockpile would result in an eventual OPV restart due to the lack of a viable alternative oSIA vaccine to prevent ultimate exportations of the outbreak virus to other populations with low immunity levels. Thus, an insufficiently large stockpile carries some risk of ultimately leading to OPV restart (i.e., an insufficient stockpile may lead to much higher OPV demands associated with OPV restart).

The average numbers of cases for each policy show a clear dichotomy between iterations that typically control outbreaks rapidly and iterations that led to OPV restart (Table 5). Iterations with controllable outbreaks represent the most common outcome and yielded fewer than 1,000 expected cases on average between OPV cessation of each type and the end of the analytical time horizon. IPV10 instead of IPV5 reduced the expected average number of post-OPV-cessation cases in those iterations by more than half from 340 to 120, while No IPV more than doubled the expected average number of cases to 840. IPV through T_end_ increased the expected average number of cases with no OPV restart compared to IPV5 or IPV10, because in 4 iterations transmission resulting from a late release from an sIPV site continued until T_end_ (without resulting in an OPV restart), which drove up the average. Iterations that restart OPV typically involve one year with a very high burden of paralytic cases followed by all remaining years with either high burden (i.e., OPV restart without SIAs) or low burden (i.e., OPV restart with SIAs). With both options, for runs with OPV restart, the year prior to the OPV restart drives the average number of cases to over 100,000 cases. Despite the relative rarity of OPV restarts, they significantly increase the expected average cases. Nevertheless, the expected average cases for all OPV cessation policies remain far below the expected cases of RC no SIAs, which results in an ongoing VAPP burden in all OPV-using populations and re-established cVDPV transmission in many of them.

**Fig. 1:**
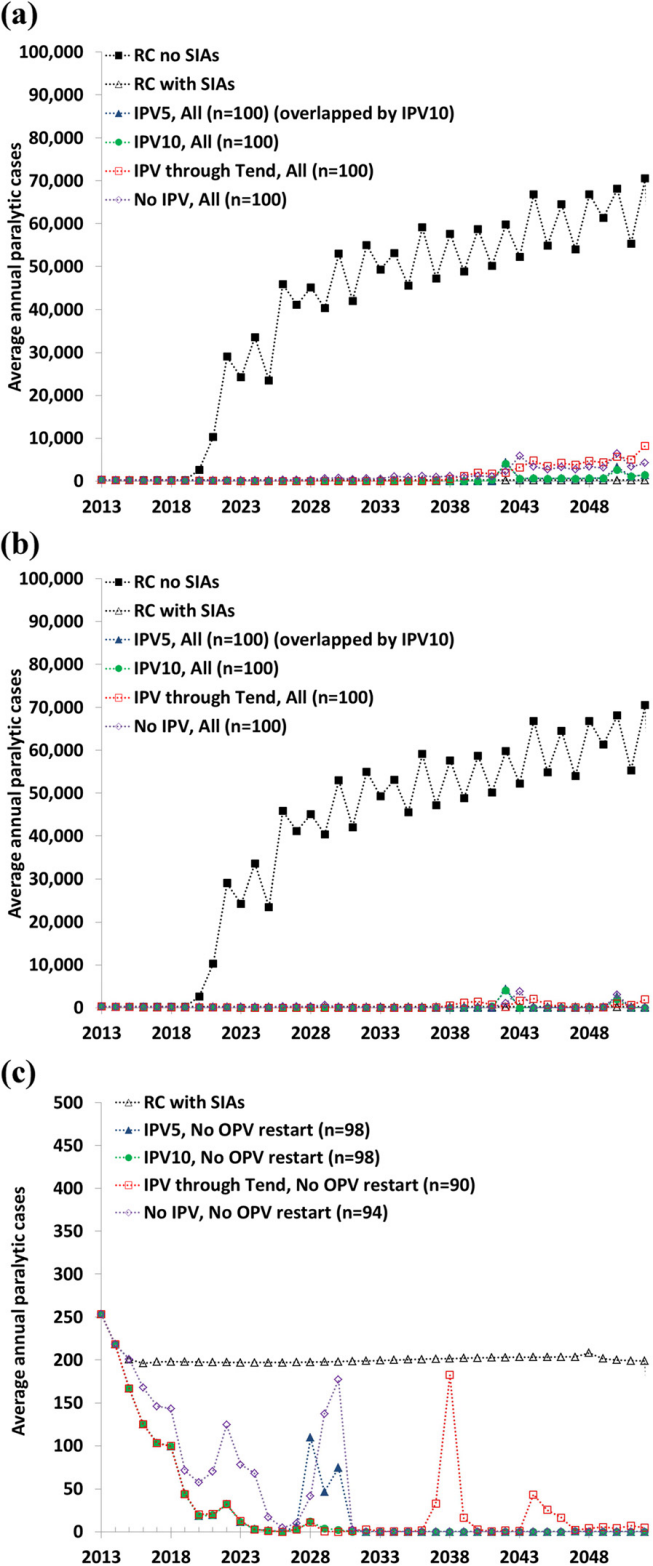
Expected, undiscounted burden of polio cases for the main policy options based on 100 stochastic iterations of the global poliovirus transmission model. (a) Assuming OPV restart without SIAs if more than 50,000 cumulative cases occur after. (b) Assuming OPV restart with SIAs if more than 50,000 cumulative cases occur after 2016. (c) Including only iterations without OPV restart (omitting reference case without SIAs, which remains beyond the scale for this panel). See Table 1 for policy abbreviations.

Figure 1 shows the average annual incidence (i.e., undiscounted cases) for the full analytical time horizon based on 100 stochastic iterations of the model for the different policies. Clearly, RC no SIAs represents the worst option in terms of annual cases (Fig. 1a and b). On this scale, the burdens for RC with SIAs and all OPV cessation options remain negligible until the time when larger, uncontrolled outbreaks occur in a small number of iterations, resulting in bumps in the average numbers of cases. Subsequent years either exhibit noticeable average numbers of cases (i.e., Fig. 1a assuming OPV restart without SIAs) or very small numbers of cases (i.e., Fig. 1b assuming OPV restart with SIAs) compared to the reference case without SIAs. To better show the behavior for typical runs that do not involve uncontrolled outbreaks, Fig. 1c omits RC no SIAs and all iterations that lead to an OPV restart. For IPV5, the dynamics in Fig. 1c reflect the average incidence from relatively common but small outbreaks between OPV2 cessation in 2016 and the end of both IPV use for RI and mOPV use for oSIAs in 2024. The average numbers of cases in subsequent years reflect lower probability events with higher consequences due to iVDPV introductions in relatively medium- or low-R_0_ populations that get controlled by IPV oSIAs and/or local burn-through of susceptible individuals before the virus can spread more widely, resulting in more peaky behavior associated with larger outbreaks in a few stochastic iterations. IPV10 substantially reduces the expected probability and consequences of these events compared to IPV5. However, for IPV through T_end_, Fig. 1c shows the occurrence of some late releases of Sabin seed strains from IPV production sites, including 4 iterations in which LPV transmission continued until T_end_ without triggering an OPV restart. Despite the possibility of outbreaks after OPV cessation, the expected number of annual cases for the subset of typical runs that do not result in an OPV restart remains below the expected annual number of VAPP cases of RC with SIAs in each year, and very far below the expected annual burden of RC without SIAs (off-scale in Fig. 1c).

### Expected future vaccination costs

**Fig. 2:**
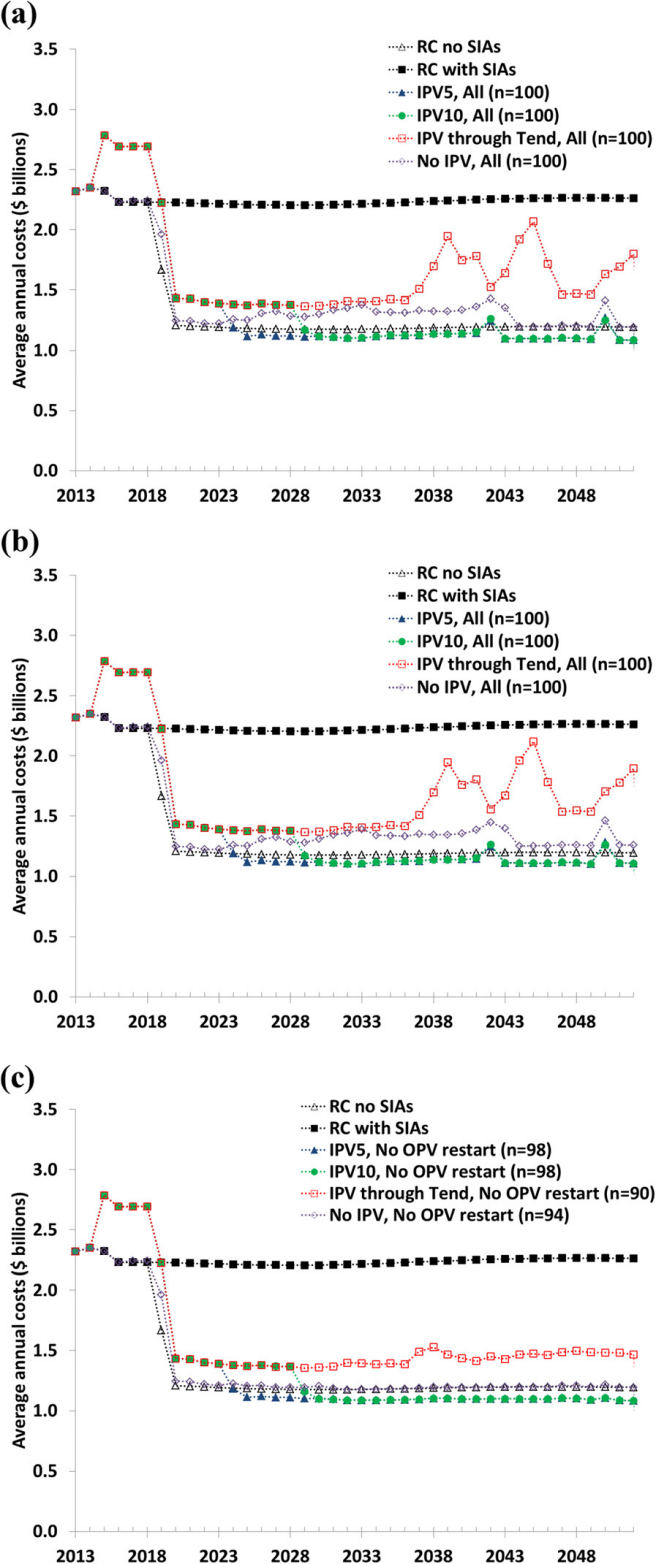
Expected, undiscounted vaccination costs in 2013 United States dollars ($) for the main policy options based on 100 stochastic iterations of the global poliovirus transmission model. (a) Assuming OPV restart without SIAs if more than 50,000 cumulative cases occur after 2016). (b) Assuming OPV restart with SIAs if more than 50,000 cumulative cases occur after 2016. (c) Including only iterations without OPV restart. See Table 1 for policy abbreviations.

Figure 2 shows the expected vaccinations costs over time, which do not include the global programmatic costs associated with preparing for OPV cessation or any specific risk management activities after OPV cessation other than RI and oSIAs. The panels break down the results by OPV restart outcome similar to the panels of Fig. 1. The first years show a marked increase in vaccination costs associated with the introduction of IPV in RI. For all OPV cessation options the costs drop dramatically after OPV13 cessation in 2019, which coincides with the cessation of SIAs for RC without SIAs. A further drop in costs occurs at the time of cessation of universal IPV use (if applicable). Nevertheless, significant costs continue to occur throughout the analytical time horizon due to the assumed continued use of IPV in RI in UMI and HIGH blocks that already use IPV at T_0_. For IPV through T_end_, the continued IPV use everywhere combined with the need to respond to releases of Sabin seed strains from sIPV production sites in some iterations imply much higher costs than the other OPV cessation policies and RC no SIAs, but these costs remain below the expected costs of RC with SIAs. The average costs of No IPV highlight the important costs associated with oSIAs after OPV cessation, for which we assume relatively higher costs than pSIAs, particularly for repeated and widespread but relatively ineffective IPV oSIAs during years when we assume mOPV no longer represents an option for oSIAs. Given that this occurs in some iterations, the reactive behavior associated with No IPV results in higher expected vaccination costs than preventive investments in IPV RI with IPV5 or IPV10. Notably, the No IPV option, thus, includes outbreak response with IPV when it represents the only polio vaccine option, including use in countries that do not use IPV in RI.

### Economic analyses

Table 6 summarizes the results of the economic analysis for IPV5 compared to the two RCs, broken down by income level and aggregated over the 40-year time horizon. In HIGH blocks, IPV5 implies a different path than the RCs in only 2 out of 10 blocks, which would switch to IPV-only after OPV13 cessation as opposed to continuing sequential IPV/OPV use in the RCs (Table 1). This change results in a relatively small increase in costs, but if divided by a very small number of VAPP cases prevented yields very high ICER of approximately $3-5 million per DALY averted, consistent with the known high societal willingness-to-pay to prevent VAPP in developed countries that already made the switch from OPV to IPV [60]. In UMI blocks, the expected cases similarly reflect VAPP cases, which decrease with the move to an eventual IPV-only schedule, but at a high incremental cost of $12 billion (and thus high ICERs), particularly compared to the costs of RC no SIAs. The impact of switching to IPV in UMI and HIGH blocks appears much less dramatic if expressed in terms of INBs, with relatively small negative INBs, except for the comparison of IPV5 to RC no SIAs in UMI blocks, which yields INBs of $-3.5 billion and significantly decreases the positive global INBs. LOW and LMI countries appropriately remain the focus of attention, because the impact of the policy choices on costs and cases remains much more significant.

**Table 6:** Economic analysis results in 2013 United States dollars for IPV5 compared to both reference cases (RCs) involving continued OPV use

Income level	Discounted, cumulative vaccination costs ($ billions)		Discounted, cumulative paralytic polio cases		Incremental costs ($ billions)	Paralytic polio cases prevented	Incremental cost-effectiveness ratio (ICER)		Incremental net benefits (INBs) ($ billions)
	IPV5	Reference case	IPV5	Reference case			Per paralytic polio case prevented ($/case)	Per DALY averted ($/DALY)	
IPV5 vs. RC no SIAs and OPV restart without SIAs									
LOW	2.9	3.9	2,700	420,00	−1.2	420,000	CLS	CLS	4.7
LMI	6.1	7.4	3,700	350,00	−3.6	350,000	CLS	CLS	15
UMI	12	8.1	150	1,200	3.7	1,000	3,600,000	250,000	−3.5
HIGH	16	15	3	8	0.4	5	80,000,000	5,600,000	−0.4
World	37	35	6,500	770,000	−0.6	770,000	N/A	N/A	16
IPV5 vs. RC with SIAs and OPV restart with SIAs									
LOW	2.9	8.9	1,400	1,500	−6.0	180	CLS	CLS	6.0
LMI	6.1	18	2,400	2,400	−12	−48	CSLC	CSLC	12
UMI	12	11	150	920	0.7	780	870,000	62,000	−0.5
HIGH	16	16	3	8	0.2	5	41,000,000	2,900,000	−0.2
World	37	53	3,900	4,800	−17	910	N/A	N/A	17

*Abbreviations* *(see Table* 1 *for policy abbreviations):* CLS, cost- and life-saving; CSLC, cost-saving but life-costing; DALY, disability-adjusted life-year; HIGH, high-income; ICER, incremental cost-effectiveness ratio; INBs, incremental net benefits; LMI, lower middle-income; LOW, low-income; N/A, not applicable; OPV, oral poliovirus vaccine; SIA, supplemental immunization activity; UMI, upper middle-income

Table 6 suggests that RC no SIAs results in many more cases than expected with IPV5. Despite the inclusion of 1 IPV dose in RI during 2015–2024 with IPV5, RC no SIAs remains more costly in LOW and LMI blocks. Consequently, IPV5 represents a cost-and life-saving option compared to RC no SIA, yielding almost $20 billion in INBs in the combined LOW and LMI blocks. If instead we compared IPV5 in LOW and LMI blocks with RC with SIAs, then Table 6 suggests significant savings with a small incremental number of expected cases (LOW blocks) or a small number of expected prevented cases (LMI blocks, not visible with the two significant digits shown in Table 6). Consequently, we find that compared to RC with SIAs, IPV5 represents a cost- and life-saving policy in LOW blocks and a cost-saving but life-costing policy in LMI blocks. Unlike the ICERs that change dramatically with small denominators, the INBs remain more robust to the choice of RC, with the difference in vaccination costs for OPV with SIAs roughly equivalent to the societal costs of paralytic polio cases associated with RC no SIAs. The global INBs amounts to approximately $16 billion or $17 billion for comparison to RC no SIA or RC with SIAs, respectively. The negative INBs in UMI and HIGH blocks reduce the global INBs, which equal $18-20 billion if we exclude the two highest income levels.

Table 7 shows the expected global costs, cases, and INBs for the other main policy options and the modeled IPV5 variations. IPV10 increases costs compared to IPV5 while preventing a relatively small number of additional cases, which decreases the global INBs by approximately $0.8 billion. As shown above, IPV through T_end_ increases the expected number of cases while increasing costs, leading to an overall reduction of the global INBs of approximately $6 billion. No IPV does not save costs due to relatively high numbers of oSIAs required, and it also yields more expected cases compared to IPV5. Consequently, for No IPV the global INBs decrease by approximately $0.5 billion.

**Table 7:** Expected global vaccination costs, paralytic cases, and incremental net benefits in 2013 United States dollars ($) for various policy options and alternative assumptions, compared to the reference case without SIAs (i.e., policy RC no SIA)

Global minimum policy	Number of iterations with OPV restart	Expected vaccination costs ($ billions)	Expected paralytic polio cases	Expected incremental net benefits ($ billions)
IPV5	2	37	6,500	16
IPV10	2	38	6,200	15
IPV through T_end_	10	42	21,000	10
No IPV	6	37	23,000	15
Variations on IPV5				
IPV5, PAVD40%	2	37	6,500	16
IPV5, PAVD90%	1	36	2,300	16
No tOPV intensification prior to OPV2 cessation	3	37	9,200	16
Doubled frequency of exportations	5	38	29,000	14
Threshold for OPV restart				
- 15,000 cumulative cases	2	37	6,500	16
- 10,000 cumulative cases	3	37	12,000	16
- 5,000 cumulative cases	4	37	16,000	16
- 1,000 cumulative cases	8	37	48,000	15

*Abbreviations* *(see Table* 1 *for policy abbreviations)*: IPV, inactivated poliovirus vaccine; OPV, oral poliovirus vaccine; PAVD (40 %,90 %), polio antiviral drug (passive or active use, respectively); RC, reference case; SIA, supplemental immunization activity; T_end_, end of analytical time horizon (i.e., December 31, 2052); tOPV, trivalent OPV

The IPV5, PAVD40% variation only prevented a small number of outbreaks and did not notably affect the global net benefits. In contrast, active PAVD use with IPV5, PAVD90% prevented many iVDPV-associated outbreaks, including one of the two events that triggered an eventual OPV restart with IPV5 (i.e., the OPV restart caused by a long-term iVDPV excretor). Consequently, the expected number of cases for IPV5, PAVD90% decreased by almost 4,000, resulting in an expected increase in the global INBs of approximately $0.5 billion, which provides some economic justification for significant investment (e.g., $500 million) in the development of PAVDs and strategies for actively identifying and managing iVDPV excretors. The PAVD90% scenario also decreased the number of stochastic iterations for which the estimated number of mOPV oSIA doses required from the stockpile exceeded 100 million for at least one serotype from 32 to 6, and the number of stochastic iterations for which the estimated number of mOPV oSIA doses needed from the stockpile exceeded 500 million for at least one serotype from 2 to 1.

No tOPV intensification prior to OPV2 cessation led to a cVDPV2 outbreak in all 100 stochastic iterations as a result of insufficient population immunity to serotype 2 poliovirus transmission in one subpopulation at the time of OPV2 cessation. Aggressive outbreak response with 4 block-wide oSIAs controlled the cVDPV2 outbreak in all 100 stochastic iterations, leading to a relatively modest increase in average expected cases from the cVDPV2 outbreak. However, No tOPV intensification also affects population immunity to serotype 2 transmission in other subpopulations which would otherwise intensify tOPV use. Consequently, in one of the 100 stochastic iterations, we observed a different realization of exportations related to an iVDPV2-associated outbreak that ultimately led to an OPV restart. Thus, failure to intensify tOPV use prior to OPV2 cessation resulted in a notable increase in the average expected number of cases by almost 3,000, and the need for mOPV2 use to respond to the cVDPV2 outbreak that increased the expected average costs by approximately $0.2 billion and decreased the global INBs by $0.3 billion. Failure to intensify tOPV use prior to OPV2 cessation did not affect the number of stochastic iterations for which the estimated number of mOPV oSIA doses exceeded 500 million for at least one serotype. However, the cVPDV2 outbreaks significantly increase expected demand from the stockpile by almost 120 million mOPV2 doses, which exceeds the size of the currently planned filled mOPV2 stockpile.

The assumed speed of viral spread between subpopulations affects the ability of aggressive oSIAs to control outbreaks after OPV cessation, prevent further spread, and avoid eventual OPV restarts. For IPV5, we explored the impact of changing the exportation threshold E* from 200,000 to 100,000 CEIs, which effectively doubles the frequency of exportations. For IPV5, this change resulted in 3 additional iterations with an eventual OPV restart and typically larger outbreaks in iterations without an eventual OPV restart. As shown in Table 7, the increased frequency of exportations leads to higher expected costs, more expected cases, and a decrease in the global INBs by approximately $1.2 billion compared to IPV5. This analysis demonstrates the importance of the uncertainty associated with the potential for international spread in an unprecedented world with no recent LPV exposure. Table 7 further shows the absence of any impact of varying the model choice for the threshold for restarting OPV between 50,000 and 15,000 cumulative cases, with only a small reduction in INBs for a reduction in the threshold to as low as 1,000 cumulative cases. The effect remains small because uncontrolled outbreaks typically include many thousands of cases in the year before OPV restart, so that crossing the threshold typically occurs in the same year regardless of the choice of threshold. Thus, our economic results remain robust to realistic choices for this threshold.

## Discussion

The integrated, global dynamic poliovirus transmission and stochastic risk model may help inform policy discussions and choices, but the actual choices will depend on many additional operational, political, epidemiological, and financial considerations. We estimate expected INBs of over $15 billion for a finite period of globally recommended IPV use in all countries after global OPV cessation compared to continued OPV use. This result reflects successful OPV cessation for nearly all of the model iterations, assuming a well-managed, coordinated OPV cessation process. In addition, it reflects the reality that continued OPV use implies either very high costs forever (i.e., for OPV with SIAs) or very high cases forever (i.e., for OPV without SIAs), or some outcome within this spectrum that remains approximately equivalent in terms of INB due to the high societal costs of paralytic poliomyelitis [61]. These INB results confirm the economic benefits of global polio eradication and subsequent OPV cessation reported by prior economic analyses [26, 40, 61–65]. The quantitative results differ from prior analyses because of differences in the framing of the analyses and evolving policies and assumptions. For example, our prior analysis of post-eradication policies [26] considered a 20-year period after simultaneous cessation of all three OPV serotypes instead of the 40-year time horizon encompassing phased OPV cessation in this analysis. The prior analysis [26] found negative INBs for indefinite IPV use compared to OPV without SIAs and encouraged research to develop more affordable IPV, which contributes to the positive INBs observed in this analysis. Our prospective economic analysis finds similar positive INBs going forward compared to an analysis [40] that found $13-23 billion (year 2010 United States dollars) in INBs for 2013–2035 when comparing global polio eradication in mostly low- and lower middle-income countries with a counterfactual policy of relying only on RI since 1988. The current model includes much more complexity than any prior integrated economic model, including transmission between populations, increased immunity states to characterize population immunity and waning, OPV evolution to simulate cVDPV emergence, serotype differences, and a detailed model to estimate iVDPV prevalence after OPV cessation [54].

Similar to prior analyses, characterizing incremental cost-effectiveness at a global level remains challenging because we cannot aggregate ICERs across income levels due to different criteria as to what constitutes a cost-effective intervention in different income levels [26, 27, 39, 45] In this analysis, the ICERs across income levels range from highly cost- and life-saving in the lower income levels to relatively cost-ineffective (i.e., compared to other public health interventions) due to very small denominators in the higher income levels that at this point only benefit from VAPP reduction. Cost-effectiveness analyses from specific high- and upper middle-income countries similarly estimate high costs per prevented polio case [60, 66–68], and the reality that countries nevertheless chose IPV over OPV suggest a high societal willingness-to-pay to prevent VAPP cases.

In addition to providing some health economic justification for IPV use during the endgame, the model provides several important insights related to its role. First, the model confirms that IPV provides only a limited reduction in cVDPV risks after OPV cessation, because the same conditions that favor cVDPV outbreaks after OPV cessation also limit the impact of IPV RI on population immunity to poliovirus transmission [24]. Second, despite the limited impact of IPV on cVDPV risks, global IPV use substantially reduces medium and long-term risks. This occurs because we anticipate most of the medium- and long-term poliovirus reintroduction risk (i.e., from long-term iVDPV excretors and unintentional or intentional releases from laboratory containment failure) to come from populations characterized by relatively lower R_0_, lower contribution to transmission from fecal-oral spread, and higher RI coverage. In these populations, IPV may prevent the initial transmissions that lead an introduction to establish population-wide transmission. Thus, although No IPV saves considerable costs initially, our results suggests that it ultimately leads to higher expected costs due to the need to respond to more and larger outbreaks and the increased probability of failing to control outbreaks. Third, continued IPV use everywhere may present a different risk associated with the possible production of IPV in high-R_0_ populations if sIPV production occurs in these areas. The model suggests that even Sabin IPV seed strains may establish transmission if released in high-R_0_ populations. Fourth, based on the current evidence [20–22, 24] our model suggests that in high-R_0_ populations, even aggressive outbreak response using IPV likely will not stop transmission long after OPV cessation. Thus, no viable outbreak response strategy would exist to stop poliovirus spread if it occurs more than approximately 5–10 years after OPV cessation and in the absence of a large mOPV stockpile in populations with conditions conducive to fecal-oral poliovirus transmission. The use of mOPV for oSIAs longer after OPV cessation, while able to effectively control the outbreak, may spread to other populations outside the response with low enough population immunity to support transmission of OPV-related viruses and/or may create new long-term iVDPV excretors.

As previously demonstrated [52], the prevention of cVDPVs after OPV cessation requires intense SIAs with homotypic OPV prior to OPV cessation. This analysis suggests that prevention represents the best risk management strategy. However, on the current path, the high probability of at least one outbreak after OPV cessation and the small but non-zero probability of uncontrolled outbreaks underscore the importance of numerous risk management efforts in addition to IPV use for successful OPV cessation and beyond.

First, aggressive outbreak response plans should represent a prerequisite for OPV cessation, because they can make the difference between experiencing only controlled outbreaks and failing to control outbreaks leading to OPV restart. Our model includes sufficiently aggressive outbreak response to minimize the probability of failing to control outbreaks, ranging from a minimum of 4 initial rounds in populations with approximately 10 million people to a maximum of 6 initial rounds in populations with approximately 100 million people (Table 4), in a few cases repeated multiple times to ultimately interrupt transmission. Further research may determine whether less aggressive outbreak response may suffice in some populations. Development of a clear strategy to determine the scope of outbreak response after OPV cessation remains a critical area of research, including the choice of vaccine (IPV, mOPV, tOPV) and consideration of the quality of rounds, response delays, geographical scope, and interval between rounds.

Second, in view of the problems associated with currently available vaccines to respond to any outbreaks long after OPV cessation, development of new poliovirus vaccines with the ability of OPV to induce intestinal immunity but without its risks (e.g., OPV that does not revert to VDPV or IPV that provides intestinal immunity, all produced with non-replicating strains) could greatly reduce the long-term risks. In the context of the possibility of high-consequence events associated with release of live poliovirus seed strains used for IPV production, efforts to develop non-replicating IPV seed strains may prove very valuable if countries or the world collectively intend to continue using IPV for many years.

Third, high bio-containment levels of laboratories and any IPV production sites that use any replicating seed strains in medium- to high-R_0_ countries remain important, regardless of the level of RI coverage with IPV in the surrounding population given that fecal-oral transmission can readily occur despite high IPV-only coverage [22, 23]. Environmental surveillance in populations surrounding IPV production sites that use replicating seed strains may offer a complementary strategy to help decrease the time until detection of any release compared to AFP surveillance, which may improve the chances of controlling the outbreak before extensive spread, although doing so requires a viable long-term outbreak response strategy in addition to early detection. In the absence of such as strategy and given that IPV production sites experienced multiple accidental LPV releases in the past despite high bio-containment levels [55, 56], our model results suggest the need to discourage production of IPV using any replicating seed strains beyond the first few years after OPV cessation in medium- to high-R_0_ settings.

Fourth, high-quality surveillance represents an essential ingredient for successful OPV cessation. This analysis assumed only case-based surveillance, but future analyses may consider the benefits of different levels of environmental surveillance (e.g., a global system focused on high-risk areas) as well as the consequences of reduced AFP surveillance quality over time.

Fifth, our results demonstrate that world health leaders should expect small outbreaks and the need to use some vaccine from the stockpile aggressively to prevent subsequent wider spread, which demonstrates that creation of the vaccine stockpile represents a prerequisite for OPV cessation [15, 69]. Depending on the required scope of outbreak response needed to contain the outbreak and the time for filling from bulk, the stockpile may require more filled mOPV doses than currently planned for one or more serotypes, and planning for a global IPV stockpile should start as soon as possible. Vaccine stockpile needs require further analysis and consideration in the context of outbreak response plans.

Finally, our model reveals potential value of PAVDs, as long-term iVDPV excretors emerge as the principal source of outbreaks after OPV cessation (i.e., assuming no cVDPV outbreaks due to tOPV intensification prior to OPV2 cessation and continued bOPV SIAs through OPV13 cessation). For effective PAVD use, efforts to identify, treat, and manage asymptomatic long-term iVDPV excretors appear as important as efforts to develop effective PAVDs.

As with any model, our analysis comes with some limitations. Although the DEB model reflects extensive expert reviews of the literature [10, 20, 21] and the model calibration process involved a wide range of situations [6, 23, 24, 47, 50, 70–72], the model limitations from prior analyses [47] carry forward to the global model. The model assumes spatially-homogeneous (age-heterogeneous) mixing in subpopulations of approximately 10 million people, which implies faster spread than more heterogeneous mixing, which we attempted to counter-balance with what might appear as a relatively low assumed rate of exportations between subpopulations. Other limitations carried forward from the poliovirus transmission and OPV evolution model include uncertainty about the numerical impact of IPV-only on poliovirus transmission in different settings (which determines how fast population immunity to transmission decreases after OPV cessation), the extent with which waning of immunity affects transmission, the relatively simple age-mixing structure, the uncertain speed of OPV evolution within populations, and the construct to capture die-out in the deterministic model [47]. Specific limitations of the integrated global model include the characterization of the global variability and mixing using a finite number of subpopulations (which only approximates the true variability and global mixing patterns), the conservative assumption that R_0_ values and RI coverage levels will remain constant into the future, the exclusion of global programmatic costs for both the OPV cessation policies and continued OPV use (which may partly cancel out in the incremental outcomes, but imply underestimation of the non-incremental costs), and the uncertainties discussed below. Moreover, while our model captures the possibility of exportations of OPV used during an outbreak response to other subpopulations, it does not account for the potentially higher probability of exportation of OPV at the borders between the targeted and non-targeted population that may mix more intensely. We did not perform additional uncertainty or sensitivity analyses because the computational costs of doing so remain prohibitive and changing any of the assumptions of the poliovirus transmission and OPV evolution model would reduce its consistency with observed behavior in the modeled specific situations unless we recalibrate the entire model [47]. We also based our results on only 100 model iterations, with further iterations expected to lead to the realization of some other sequences of rare events that we did not yet observe in the model. Finally, our estimates of future vaccine prices and wastage remain uncertain and significantly impact the economic results, which suggest the need for future evaluation of these assumptions. Future studies should address uncertainties as more evidence becomes available and areas identified in this analysis as important for further work (e.g., outbreak response strategies), and consider more stochastic iterations as needed.

Despite the many complexities included in our global model, many uncertainties and stochastic events limit our ability to predict what will actually happen in an unprecedented post-OPV era and which may lead to a wide range of potential consequences. Probably the most important uncertainty relates to the speed of spread of polioviruses between populations in the absence of any recent prior LPV exposure. We explored this uncertainty by varying the threshold (i.e., E*) to trigger potentially effective exportations, which demonstrated a substantial impact on the ability to control outbreaks after OPV cessation. Measurement or other direct estimation of this model input remains impossible, and therefore we cannot know with high confidence whether the true value lies below, inside, or above the range we explored. However, comparison of the modeled behavior of cVDPV outbreaks within a year after OPV cessation with the experience from historical cVDPV outbreaks provides some indication that the range we considered probably adequately captures the kinetics and appropriately corrects for the simplification inherent in the assumption of spatially-homogeneous mixing within subpopulations in the model. In addition, exportations represent stochastic events, with chance determining the actual path. The value of E* interacts directly with the assumed relationship between population immunity to transmission and the probability of an effective introduction (i.e., PEF), which also remains uncertain. Different assumptions about the speed of spread between populations will imply different requirements for the aggressiveness of the outbreak response and stockpile size. Given the uncertainty, this analysis suggests that erring on the side of more aggressive outbreak response represents the prudent approach as long as the risk of mOPV exportations remains low (i.e., during the first few years after OPV cessation). Other key uncertainties that affect the probability of outbreaks and/or their consequences include (1) the long-term survival of immunodeficient patients in lower income levels, (2) the impact of IPV-induced immunity on transmission and/or extent of fecal-oral spread in different populations, (3) the quality and frequency of tOPV rounds until OPV2 cessation and bOPV rounds leading up to OPV13 cessation, (4) the future rate of releases of WPV or Sabin seeds strains from IPV production sites in the context of different levels of containment, (5) the unpredictable occurrence of very rare other events long after OPV cessation with very large consequences, and (6) the potential for OPV used during outbreak response to generate new VDPV outbreaks elsewhere.

## Conclusions

This analysis suggests a relatively high probability of significant economic benefits associated with OPV cessation and global poliovirus risk management efforts for the next 40 years, with a small probability of a failure to contain outbreaks after OPV cessation. The results highlight the critical importance of multiple long-term poliovirus risk management efforts and important uncertainties that remain for the post-OPV-cessation era.

## Additional file

Additional file 1:
**Technical Appendix.** (DOCX 257 kb)

## Abbreviations

AFP: Acute flaccid paralysis
bOPV: Bivalent OPV (serotypes 1 and 3)
CEI: Cumulative effective infection
CLS: Cost-and life-saving
CSLC: Cost-saving but life-costing
cVDPV(1,2,3): Circulating vaccine-derived poliovirus (serotype 1, 2, or 3, respectively)
CVID: Common variable immunodeficiency disease
DALY: Disability-adjusted life-year
DEB: Differential equation-based
DES: Discrete-event simulation
E*: Threshold number of CEIs to trigger an exportation
EPI: Effective proportion infectious
EPI*: Threshold EPI below which force-of-infection becomes zero
FRRs: GPEI financial resource requirements
GNI: Gross national income
GPEI: Global Polio Eradication Initiative
HIGH: High-income
ICER: Incremental cost-effectiveness ratio
INBs: Incremental net benefits
IPV: Inactivated poliovirus vaccine
IPV#: Global minimum policy of IPV use for # years
iVDPV: Immunodeficiency-associated vaccine-derived poliovirus
LMI: Lower middle-income
LOW: Low-income
LPV: Live poliovirus
mOPV(1,2,3): Monovalent OPV (serotype 1, 2, or 3, respectively)
N Pak/Afg: North Pakistan and Afghanistan
NID: National immunization day
oPID: Other PID
OPV: Oral poliovirus vaccine
OPV## cessation: Globally-coordinated cessation of OPV containing the serotype(s) indicated by ##
oSIA: Outbreak response SIA
PAVD(40 %,90 %): Polio antiviral drug (passive or active use policy, respectively)
PEF: Probability of effective introduction function
PID: Primary immunodeficiency disease
POL3: Coverage with 3 or more non-birth RI doses
pSIA: Planned, preventive SIA
R_0_: Basic reproduction number
RC: Reference case
RCT: Relative contribution to combined fecal-oral and oropharyngeal transmission
RI: Routine immunization
R_n_: Mixing-adjusted net reproduction number
SIA: Supplemental immunization activity
sIPV: IPV produced from Sabin seed strains
SNID: Sub-national immunization day
T_0_: Beginning of the analytical time horizon (i.e., January 1, 2013)
T_end_: End of the analytical time horizon (i.e., December 31, 2052)
tOPV: Trivalent OPV
UMI: Upper middle-income
VAPP: Vaccine-associated paralytic poliomyelitis
VDPV: Vaccine-derived poliovirus
WPV(1,2,3): Wild poliovirus (serotype 1, 2, or 3, respectively)
US$2013: 2013 United States dollars
