# Supplementary material for: An economic analysis of poliovirus risk management policy options for 2013–2052
Source: BMC Infect Dis. 2015 Sep 24;15:389. doi: 10.1186/s12879-015-1112-8 (PMC4582932; doi:10.1186/s12879-015-1112-8)
Supplement: Additional file 1: — Technical Appendix. (DOCX 257 kb) [file 12879_2015_1112_MOESM1_ESM.docx]

**TECHNICAL APPENDIX**

**Main paper: Duintjer Tebbens RJ, Pallansch MA, Cochi SL, Wassilak SGF, Thompson KM: An economic analysis of poliovirus risk management policy options for 2013-2052. BMC Infect Dis 2015;** **DOI: 10.1186/s12879-015-1112-8**

See main paper for list of abbreviations. Table numbers not preceded by “A” refer to the main paper.

***A1. Equations for ICER and INB estimation***

We express the ICER in incremental costs per prevented (paralytic) polio case and per disability-adjusted life-years (DALYs)[[1](#_ENREF_1)] averted according to the following equations:

ICER_case_ (*alt* vs. *ref*) = incremental costs per prevented polio case

= ( (FC_alt_-FC_ref_) – T×(PP_ref_-PP_alt_) ) / (PP_ref_-PP_alt_)

ICER_DALY_ (*alt* vs. *ref*) = incremental costs per averted DALY

= ( (FC_alt_-FC_ref_) – T×(PP_ref_-PP_alt_) ) / (D× (PP_ref_-PP_alt_))

where FC_ref_ = cumulative, discounted financial costs associated with the reference case

FC_alt_ = cumulative, discounted financial costs associated with the alternative policy

PP_ref_ = cumulative, discounted number of polio cases with the reference case

PP_alt_ = cumulative, discounted number of polio cases with the alternative policy

T = average treatment costs per polio case

D = average number of DALYs per polio case.

We compute the incremental net benefits of an alternative option (*alt*) compared to each reference case (*ref*) as:

INB (*alt* vs. *ref*) = (T+S) ×(PP_ref_-PP_alt_) – (FC_alt_-FC_ref_)

where S = average societal economic costs per polio case.

***A2. Description and calibration of poliovirus transmission model***

We conducted an extensive expert review[[2](#_ENREF_2), [3](#_ENREF_3)] and elicitation process[[4](#_ENREF_4)] to inform the generic model inputs (e.g., characterization of immunity states, properties of the OPV evolution process).[[5](#_ENREF_5)] We then calibrated the generic model inputs within the uncertainty ranges obtained from the expert elicitation process[[4](#_ENREF_4)] to produce behavior consistent with the evidence for 10 different epidemiological situations, which include experience with WPV incidence and die-out for all 3 serotypes, absence of cVDPVs in some situations but emergence in others, VAPP cases and secondary OPV immunity, and different age distributions of cases.[[5-7](#_ENREF_5)] To simulate die-out and cVDPV emergence in the model, we evaluate the effective proportion infectious (EPI, i.e., the infectious-weighted prevalence) for any given virus strain (i.e., WPV or any OPV reversion stage) and mixing age group to determine if it exceeds a “transmission threshold” (EPI*) that we calibrated across the situations.[[5](#_ENREF_5)] If EPI<EPI*, then the model sets the force-of-infection for the virus strain and mixing age group to 0 and transmission dies out. We further developed methods to: (1) appropriately characterize age-specific RI with OPV-only, IPV-only, or IPV/OPV, including simultaneous administration of both vaccines,[[8](#_ENREF_8)] (2) model the reality of repeatedly missed children during supplemental immunization activities (SIAs),[[6](#_ENREF_6)] and (3) characterize overall population immunity to poliovirus transmission as a single quantity in the context of partial immunity to poliovirus transmission and heterogeneous mixing between age groups and subpopulations.[[9](#_ENREF_9)]

***A3. Modeling virus reintroductions using the probability of an effective introduction function (PEF)***

We assume a logarithmic increase in PEF for R_n_>1, with values of 0.2 when R_n­_ equals 1 (i.e., population immunity at the threshold to support sustained transmission) and 0.5 when R_n_ equals 5 or more (i.e., very low population immunity), as shown in Figure A1. We associate a random uniform number (U) with every potential poliovirus introduction. If a potential introduction event occurs during a stochastic iteration of the global poliovirus transmission model, then we assume that the introduction is effective if U<PEF(R_n_). If an effective introduction occurs, then the model generates exactly enough initial infections in the receiving subpopulation to exceed the transmission threshold and potentially lead to circulation. Specifically, we increase the effective prevalence of infections (i.e., EPI) to the transmission threshold (i.e., EPI*) (see appendix A2) by moving a fraction EPI*of all fully susceptible individuals in each age group to the first infectious stage.[[5](#_ENREF_5)] If the proportion fully susceptible in an age group equals less than EPI*, then this does not preclude an effective virus introduction, because individuals with immunity to polio (disease) still contribute to varying degrees to poliovirus transmission.[[5](#_ENREF_5)] Therefore, if the proportion fully susceptible in an age group already equals less than EPI*, then we move a fraction EPI*/RCT(i) in each immunity state i to the first infectious stage for the immunity state, where RCT equals the relative contribution to combined fecal-oral and oropharyngeal transmission for the immunity state i.[[4](#_ENREF_4), [5](#_ENREF_5), [10](#_ENREF_10)]

***A4. List of subpopulation characteristics, assignment to blocks and regions, and the model mechanics of selecting the locations for virus exportations***

Table A1 provides the assumptions that we varied across the 710 subpopulations in the global model, intended to reflect real variability that exists in the world and allow the model to capture stratified mixing between subpopulations. Many subpopulation within blocks, and some blocks within large multi-block countries reflect identical properties, and therefore we showed these as single rows in Table A1. Table A2 defines the OPV take rate and SIA impact level inputs used in Table A1. To characterize the variability in conditions relevant to poliovirus transmission, we focus on a number of model inputs (i.e., the basic reproduction number (R_0_), its seasonal amplitude (*α*) and peak day (*pd*), the proportion of transmissions via the oropharyngeal route (*p^oro^*), the strength of preferential mixing (*κ*), the OPV take rate level (*trl*), RI coverage with 3 or more non-birth doses (*POL3*), the SIA impact level (*sil*, characterized by the true coverage and repeated missed probability).[[6](#_ENREF_6)] and the detection threshold (*dt*) based on prior work) (see appendix A4).[[11](#_ENREF_11), [12](#_ENREF_12), [6](#_ENREF_6), [5](#_ENREF_5)] We assume inputs not controlled by policies (e.g., R_0_, *α,* *pd*, *p^oro^_,_ κ*, and *tr*) remain the same for all subpopulations within a block, with the exception of 3 HIGH blocks in which we allowed a slightly higher R_0_ and lower *p^oro^* in one subpopulation than all other subpopulations to examine the possibility of some poliovirus spread in the areas that currently use IPV-only with high coverage.[[7](#_ENREF_7)] We assume appropriate positive correlations between higher R_0_, lower *α*, lower *p^oro^*, and lower *tr.* As appropriate, we vary the more policy-controllable inputs *POL3*, *tcl*, *sil* between subpopulations of the same block to account for real heterogeneity in RI, SIAs, and surveillance within blocks. Within blocks, we assume positive correlations between higher *POL3*, higher SIA impact, and lower *dt*. We assume that one LOW and 3 LMI blocks each contain one chronically under-vaccinated subpopulation with much lower *POL3*, SIA impact, and higher *dt* than the other subpopulations. These subpopulations can sustain poliovirus transmission despite frequent SIAs and represent the last reservoirs of indigenous WPV transmission, and in this regard they resemble the final 4 reservoirs of indigenous WPV1 transmission in the real world. We characterized these populations considering lessons learned from prior modeling.[[8](#_ENREF_8), [9](#_ENREF_9)] In two of these, we assumed improvements since 2010, while in the other two we assume intensification of efforts to eliminate WPV through increased frequency of low-impact SIAs alone. Although we only used a discrete set of *POL3* values (i.e., 0.05, 0.1, 0.3, 0.6, 0.9, and 0.98), we chose values such that the averages in blocks correspond approximately to values estimated for different populations in each income level.[[13](#_ENREF_13)] We assumed no changes in coverage levels over time going forward.

Although we do not attempt to characterize the full mixing structure between all 710 subpopulations in the world, the model accounts for randomness in virus exportations while still allowing replication of the simulation by specifying a pre-run list of 200 recipient subpopulations for each subpopulation. In this list, the probability that a recipient subpopulation resides in the same block as the exporting subpopulation equals 0.96. Each time step when the CEI in a subpopulation exceeds the E*, we introduce the exported virus into the next subpopulation on the list of the exporting subpopulation, until we reach the end of the list, at which point we start again from the beginning of the list. We use the same list for all of the policies considered in an effort to reduce the differential impact of random exportations on the results for different policies, but we use a different randomly generated list for each stochastic iteration of the overall economic model.

***A5. Details about the run-up, the assumed planned, preventive SIA (pSIA) schedules in blocks that use OPV-only or IPV/OPV, and the specific vaccines used***

Facing high computational demands and highly variable polio control histories in different countries, we used a simplified and compressed run-up. We start all subpopulations at effectively 43 years before T_0_ (i.e., year -43) with only fully susceptible individuals and then introduce a single infection in each of them. In the absence of vaccination, this leads all subpopulations to a state near the oscillating pre-vaccine equilibrium after a period of time, depending on R_0_ and the seasonal amplitude. We start tOPV RI with SIAs in HIGH blocks first, effectively 23 years before T_0_, according to the subpopulation-specific *POL3* and SIA impact assumptions and apply the SIA assumptions specified in Table 4. In all other blocks, we assume tOPV RI starts effectively 13 years before T_0_, with linearly increasing *POL3* from 0 when introduced to 50% within 5 years. At that point, tOPV SIAs start at the subpopulation-specific impact level and *rcov* changes to the subpopulation-specific values as well, which in a few subpopulations with very low *POL3* implies a decrease in *POL3* due to increased reliance on SIAs for polio immunization. Blocks in different strata of income level and polio vaccine use at T_0_ (Table 3) then switch to IPV-only or IPV/OPV at different times (Table 1). We do not characterize the relatively brief period of mOPV use for some SIAs, but instead introduce bOPV for SIAs 3 years before T_0_ by switching a fraction of SIAs from tOPV to bOPV from that point forward. Table A3 shows the assumed planned, preventive SIA (pSIA) schedules in blocks that use OPV-only or IPV/OPV, depending on the WPV elimination status and RI coverage. We define WPV elimination in a block as lack of transmission of any WPV serotype in any subpopulation as a result of WPV prevalence of infections falling below the EPI*. While WPV importations can potentially delay WPV elimination, once a block reaches post-WPV-elimination status, it retains this status even after any subsequent WPV importations. All OPV-using blocks start with two annual tOPV SIAs and increase the frequency by one each year until they reach WPV elimination. In the event of continued indigenous WPV transmission in a block beyond the bOPV introduction time (i.e., T_0_-3), the vaccine choice for each round depends on RI coverage. After WPV elimination, blocks switch to the post-WPV-elimination schedule, for which the number of rounds also depends on RI coverage. All blocks that use IPV-only at T_0_ achieve WPV elimination before they switch to IPV-only, and we assume no SIAs in blocks that use IPV-only. The choice of vaccine depends on the time period, with tOPV-only initially, predominantly bOPV with some tOPV in lower RI coverage blocks after bOPV introduction, predominantly tOPV with some bOPV starting the last full year before OPV2 cessation in April 2016, and bOPV-only between OPV2 cessation and OPV13 cessation.

Figure A2 shows the results burn in period. Prior to the start of vaccination (i.e., year -23 in HIGH blocks, year -13 in other block), the global incidence oscillates around 500,000 annual cases. With RI vaccination increasing to current levels in all countries, the annual incidence drops to around 100,000 annual cases (i.e., year -9), and with the onset of SIAs (i.e., year -8) in non-HIGH blocks, the incidence drops dramatically. Global WPV2 elimination occurs within 2 years of this instantaneous global start of SIAs with most countries eliminating all indigenous WPVs within a few years. However, due to the inclusion of some blocks with properties similar to the last known WPV reservoirs with preferentially mixing under-vaccinated subpopulations (characterized by prior studies that modeled 3 of the 4 of these reservoirs[[6](#_ENREF_6), [5](#_ENREF_5)]), WPV3 continues to circulate in the last subpopulation until the start of year 1, and WPV1 until the start of year 3. In the last subpopulation with endemic WPV, a cVDPV2 also emerges, but gets controlled due to the intensification of tOPV SIAs between year 2 and OPV2 cessation.

***A6. Generation of new long-term iVDPV excretors resulting from any mOPV use after OPV cessation***

We use the DES model[[14](#_ENREF_14)] that tracks the prevalence of and generates potentially effective introductions from, long-term excretors infected prior to OPV cessation (see Methods section of the main paper) also to create new iVDPV excretors as a result of any mOPV use to respond to outbreaks after OPV cessation. For each individual born with a pre-disposition of developing a PID, the DES model pre-determines at birth whether the individual could acquire a long-term iVDPV infection if infected with OPV after PID onset.[[14](#_ENREF_14)] All patients living with a PID for any time after OPV cessation and pre-determine to exhibit potential long-term iVDPV excretion if infected could develop long-term excretion as a result of any mOPV use after OPV cessation in their subpopulation. Therefore, for all individuals meeting those criteria in a stochastic realization of the DES model, we record events that affect their ability to become newly generated iVDPV excretors, including (1) time of PID onset, (2) time of PID diagnosis (which affects their probability of receiving OPV or becoming secondarily exposed to OPV), (3) time when any intravenous immunoglobulin treatments starts or falls into a lapse (which affects their probability of establishing a long-term infection), (4) time of death, (5) times of contacts with the general populations. Moreover, we pre-determine random characteristics of any new OPV infections that these individuals would experience in the event of mOPV oSIAs in their subpopulation. To do so, we generate random numbers to characterize 10 possible exposures to OPV after OPV cessation, including (1) ten uniform random numbers to compare against the probability of infection given exposure, (2) ten random durations of an infection to apply in the event a new infection, (3) ten uniform random numbers to determine if the time of VAPP onset in the event of a new infection, (4) one uniform random number to determine whether fatal VAPP occurs in the event VAPP occurs prior to the time of death in the absence of VAPP and prior to recovery from the new infection, (5) two random numbers to determine whether the patient would receive PAVDs and whether the PAVD would clear the infection in the event of a new infection and one of the two PAVD use scenarios. In the very unlikely event of more than ten OPV exposures after OPV cessation, we restart from the beginning of the appropriate lists of random numbers. If an OPV oSIA occurs after OPV cessation, then we calculate the probability of infection given exposure for each alive, clinical PID patient not already infected at the time of the oSIA based on its diagnosis and treatments status based on infection probabilities used in the DES model.[[14](#_ENREF_14)] We then model a new infection if the random uniform number of the corresponding exposure is less than the probability of infection given exposure. If a new infection occurs, then use the infection duration of the corresponding exposure and similarly determine whether (fatal) VAPP occurs and PAVD treatment clears the infection (which depends on the PAVD scenario) by comparing the appropriate random uniform number to the appropriate probability.

***A7. Blocks with potential IPV production sites***

### The overall rate of releases from IPV production sites remains 1 per 5 years (Table 4), and for each release we randomly select the IPV production site that releases either WPV (if the production site makes wIPV) or Sabin (if the production site makes sIPV). The exact locations of future IPV production sites remains uncertain, but some indication exists about the spectrum of properties of the populations currently considering the potential to host an IPV production site in the future. Therefore, we construct a single list of potential populations that may host an IPV production site in the future and randomly select locations for each stochastic iteration from these populations as follows (see Table A1 for the properties of the blocks):

### 5 wIPV production sites deterministically distributed in 5 different subpopulations of 2 European blocks (i.e.., subpopulations 5, 6, and 7 of block 45 and subpopulations 3 and 4 of block 45

### 1 sIPV production site in a random subpopulation in the region of China and neighbors, comprising 14 blocks

### 3 more sIPV production sites in 3 different randomly selected UMI blocks from the sublist containing blocks 35, 37, 60, 62, 70 and one of blocks 61 and 63 with equal chance.

### 1 sIPV production site in a random subpopulation in the Indian region, comprising 12 blocks

### 1 sIPV production site in a random subpopulation in blocks 13, 14, or 15 with equal probability

### 1 sIPV production site in a random subpopulation of block 6 or 69 with equal probability

### For the policy option of IPV through T_end_, we add 3 more sIPV production sites to the list in the event of a release after 2029 (i.e., more than 10 years after OPV13 cessation):

### 2 in randomly selected non-Chinese UMI blocks from the list (second bullet above) that do not already have an sIPV production site

### 1 in either block 34 or in whichever of blocks 6 or 69 (last bullet above) does not already have an sIPV production site

### To ensure fair comparisons between policies, we pre-select all introductions over the entire time horizon at the beginning of a stochastic iteration. If random selection of the release location results in a LMI production site, then the release does not take place after global IPV use stopped, because LOW and LMI blocks will stop using IPV by then (while UMI and HIGH blocks continue to use IPV). This implies that the effective rate of releases goes down slightly after global IPV cessation (i.e., some fraction of releases does not occur).

***A8. Details of stochastic iterations that led to OPV restart***

Table A4 lists the characteristics of stochastic iterations that led to an OPV restart (based on 50,000 cumulative cases between 2016-2051) for one or more of the main policies.

**Table A1: List of variability inputs for 710 subpopulations in the global poliovirus transmission model (list of symbols provided at bottom of table)**

| **Region** | **Block** | **Sub-**  **popul-ation(s)** | **Income level** | **Polio vaccine at T_0_** | **R_0_** | α | **pd** | κ | **trl** | **POL3** | **sil** | **dt** | **p^oro^** |
| --- | --- | --- | --- | --- | --- | --- | --- | --- | --- | --- | --- | --- | --- |
| Africa | 1 | 1-2 | LOW | OPV-only | 11 | 0.15 | 0 | 0.35 | 3 | 0.3 | 3 | 3 | 0.3 |
| Africa | 1 | 3-3 | LOW | OPV-only | 11 | 0.15 | 0 | 0.35 | 3 | 0.6 | 4 | 3 | 0.3 |
| Africa | 1 | 5-9 | LOW | OPV-only | 11 | 0.15 | 0 | 0.35 | 3 | 0.9 | 4 | 2 | 0.3 |
| Africa | 1 | 10 | LOW | OPV-only | 11 | 0.15 | 0 | 0.35 | 3 | 0.98 | 5 | 1 | 0.3 |
| Africa | 2 | 1 | LOW | OPV-only | 10 | 0.1 | 180 | 0.35 | 3 | 0.3 | 4 | 2 | 0.3 |
| Africa | 2 | 2 | LOW | OPV-only | 10 | 0.1 | 180 | 0.35 | 3 | 0.6 | 4 | 2 | 0.3 |
| Africa | 2 | 3-10 | LOW | OPV-only | 10 | 0.1 | 180 | 0.35 | 3 | 0.9 | 4 | 2 | 0.3 |
| Africa | 3 | 1 | LOW | OPV-only | 10 | 0.15 | 180 | 0.35 | 3 | 0.3 | 3 | 3 | 0.3 |
| Africa | 3 | 2 | LOW | OPV-only | 10 | 0.15 | 180 | 0.35 | 3 | 0.6 | 3 | 3 | 0.3 |
| Africa | 3 | 5-8 | LOW | OPV-only | 10 | 0.15 | 180 | 0.35 | 3 | 0.9 | 4 | 2 | 0.3 |
| Africa | 3 | 9 | LOW | OPV-only | 10 | 0.15 | 180 | 0.35 | 3 | 0.98 | 4 | 1 | 0.3 |
| Africa | 4 | 1-2 | LOW | OPV-only | 9 | 0.2 | 0 | 0.35 | 3 | 0.6 | 4 | 2 | 0.3 |
| Africa | 4 | 3-8 | LOW | OPV-only | 9 | 0.2 | 0 | 0.35 | 3 | 0.9 | 4 | 1 | 0.3 |
| Africa | 4 | 9-10 | LOW | OPV-only | 9 | 0.2 | 0 | 0.35 | 3 | 0.98 | 4 | 1 | 0.3 |
| Africa | 5 | 1 | LOW | OPV-only | 10 | 0.1 | 120 | 0.4 | 3 | 0.3 | 3 | 3 | 0.3 |
| Africa | 5 | 2-4 | LOW | OPV-only | 10 | 0.1 | 120 | 0.4 | 3 | 0.6 | 3 | 2 | 0.3 |
| Africa | 5 | 5-10 | LOW | OPV-only | 10 | 0.1 | 120 | 0.4 | 3 | 0.9 | 4 | 1 | 0.3 |
| Africa | 6 | 1-4 | LMI | OPV-only | 9 | 0.2 | 0 | 0.4 | 2 | 0.9 | 4 | 2 | 0.3 |
| Africa | 6 | 5-6 | LMI | OPV-only | 9 | 0.2 | 0 | 0.4 | 2 | 0.9 | 4 | 1 | 0.3 |
| Africa | 6 | 7-10 | LMI | OPV-only | 9 | 0.2 | 0 | 0.4 | 2 | 0.98 | 5 | 1 | 0.3 |
| Africa | 7 | 1 | LMI | OPV-only | 7 | 0.15 | 0 | 0.4 | 4 | 0.3 | 4 | 2 | 0.3 |
| Africa | 7 | 2-8 | LMI | OPV-only | 7 | 0.15 | 0 | 0.4 | 4 | 0.6 | 4 | 2 | 0.3 |
| Africa | 7 | 9-10 | LMI | OPV-only | 7 | 0.15 | 0 | 0.4 | 4 | 0.9 | 4 | 1 | 0.3 |
| Africa | 8 | 1 | LMI | OPV-only | 8 | 0.1 | 120 | 0.4 | 3 | 0.05 | 1 | 3 | 0.3 |
| Africa | 8 | 2-10 | LMI | OPV-only | 8 | 0.1 | 120 | 0.4 | 3 | 0.3 | 3 | 2 | 0.3 |
| Africa | 9 | 1-6 | LMI | OPV-only | 9 | 0.05 | 120 | 0.4 | 3 | 0.6 | 4 | 2 | 0.3 |
| Africa | 9 | 7-10 | LMI | OPV-only | 9 | 0.05 | 120 | 0.4 | 3 | 0.9 | 4 | 1 | 0.3 |
| Africa | 10 | 1 | LMI | OPV-only | 8 | 0.1 | 120 | 0.4 | 4 | 0.6 | 4 | 2 | 0.3 |
| Africa | 10 | 2-6 | LMI | OPV-only | 8 | 0.1 | 120 | 0.4 | 4 | 0.9 | 4 | 2 | 0.3 |
| Africa | 10 | 7-10 | LMI | OPV-only | 8 | 0.1 | 120 | 0.4 | 4 | 0.98 | 5 | 1 | 0.3 |
| Africa | 11 | 1-2 | UMI | OPV-only | 7 | 0.25 | 300 | 0.35 | 5 | 0.9 | 4 | 2 | 0.6 |
| Africa | 11 | 3-10 | UMI | OPV-only | 7 | 0.25 | 300 | 0.35 | 5 | 0.98 | 5 | 1 | 0.6 |
| Australasia | 12 | 1 | LMI | OPV-only | 7 | 0.15 | 180 | 0.4 | 5 | 0.6 | 4 | 2 | 0.5 |
| Australasia | 12 | 2-10 | LMI | OPV-only | 7 | 0.15 | 180 | 0.4 | 5 | 0.9 | 5 | 1 | 0.5 |
| Australasia | 13 | 1 | LMI | OPV-only | 8 | 0.2 | 120 | 0.35 | 3 | 0.3 | 4 | 2 | 0.3 |
| Australasia | 13 | 2-7 | LMI | OPV-only | 8 | 0.2 | 120 | 0.35 | 3 | 0.6 | 4 | 2 | 0.3 |
| Australasia | 13 | 8 | LMI | OPV-only | 8 | 0.2 | 120 | 0.35 | 3 | 0.9 | 4 | 1 | 0.3 |
| Australasia | 13 | 9-10 | LMI | OPV-only | 8 | 0.2 | 120 | 0.35 | 3 | 0.98 | 4 | 1 | 0.3 |
| Australasia | 14 | 1-5 | LMI | OPV-only | 7 | 0.2 | 120 | 0.4 | 4 | 0.6 | 4 | 2 | 0.5 |
| Australasia | 14 | 6-9 | LMI | OPV-only | 7 | 0.2 | 120 | 0.4 | 4 | 0.6 | 4 | 1 | 0.5 |
| Australasia | 14 | 10 | LMI | OPV-only | 7 | 0.2 | 120 | 0.4 | 4 | 0.9 | 4 | 1 | 0.5 |
| Australasia | 15 | 1-5 | LMI | OPV-only | 6 | 0.2 | 120 | 0.4 | 5 | 0.6 | 4 | 2 | 0.6 |
| Australasia | 15 | 6-9 | LMI | OPV-only | 6 | 0.2 | 120 | 0.4 | 5 | 0.6 | 4 | 1 | 0.6 |
| Australasia | 15 | 10 | LMI | OPV-only | 6 | 0.2 | 120 | 0.4 | 5 | 0.9 | 4 | 1 | 0.6 |
| Australasia | 16 | 1-4 | HIGH | IPV-only | 5 | 0.2 | 60 | 0.45 | 7 | 0.9 | 4 | 2 | 0.8 |
| Australasia | 16 | 5-10 | HIGH | IPV-only | 5 | 0.2 | 60 | 0.45 | 7 | 0.98 | 5 | 1 | 0.8 |
| Australasia | 17 | 1-6 | HIGH | IPV-only | 4 | 0.2 | 120 | 0.45 | 8 | 0.98 | 5 | 1 | 0.9 |
| Australasia | 17 | 7-10 | HIGH | IPV-only | 4 | 0.2 | 120 | 0.45 | 8 | 0.98 | 5 | 2 | 0.9 |
| China and neighbors | 18 | 1 | UMI | OPV-only | 7 | 0.4 | 180 | 0.5 | 6 | 0.9 | 4 | 2 | 0.6 |
| China and neighbors | 18 | 2-10 | UMI | OPV-only | 7 | 0.4 | 180 | 0.5 | 6 | 0.98 | 5 | 1 | 0.6 |
| China and neighbors | 19 | 1-10 | UMI | OPV-only | 6 | 0.35 | 180 | 0.5 | 7 | 0.98 | 5 | 1 | 0.8 |
| China and neighbors | 20 | 1-10 | UMI | OPV-only | 8 | 0.35 | 180 | 0.5 | 5 | 0.9 | 4 | 2 | 0.6 |
| China and neighbors | 21-31 | 1-10 | UMI | OPV-only | 7 | 0.35 | 180 | 0.5 | 6 | 0.98 | 5 | 1 | 0.6 |
| East and Central Asia | 32 | 1 | LOW | OPV-only | 11 | 0.2 | 180 | 0.35 | 2 | 0.1 | 2 | 3 | 0.3 |
| East and Central Asia | 32 | 2-10 | LOW | OPV-only | 11 | 0.2 | 180 | 0.35 | 2 | 0.6 | 4 | 2 | 0.3 |
| East and Central Asia | 33 | 1 | LMI | OPV-only | 7 | 0.5 | 60 | 0.3 | 2 | 0.6 | 5 | 2 | 0.8 |
| East and Central Asia | 33 | 2-3 | LMI | OPV-only | 7 | 0.5 | 60 | 0.3 | 2 | 0.9 | 5 | 2 | 0.8 |
| East and Central Asia | 33 | 4-10 | LMI | OPV-only | 7 | 0.5 | 60 | 0.3 | 2 | 0.98 | 5 | 1 | 0.8 |
| East and Central Asia | 34 | 1-3 | LMI | OPV-only | 11 | 0.15 | 180 | 0.35 | 2 | 0.6 | 3 | 2 | 0.3 |
| East and Central Asia | 34 | 4-10 | LMI | OPV-only | 11 | 0.15 | 180 | 0.35 | 2 | 0.9 | 4 | 1 | 0.3 |
| East and Central Asia | 35 | 1-3 | UMI | OPV-only | 7 | 0.2 | 60 | 0.35 | 5 | 0.6 | 4 | 2 | 0.6 |
| East and Central Asia | 35 | 4 | UMI | OPV-only | 7 | 0.2 | 60 | 0.35 | 5 | 0.9 | 5 | 1 | 0.6 |
| East and Central Asia | 35 | 5-10 | UMI | OPV-only | 7 | 0.2 | 60 | 0.35 | 5 | 0.98 | 5 | 1 | 0.6 |
| East and Central Asia | 36 | 1 | UMI | IPV/OPV | 6 | 0.25 | 60 | 0.35 | 5 | 0.3 | 3 | 3 | 0.6 |
| East and Central Asia | 36 | 2 | UMI | IPV/OPV | 6 | 0.25 | 60 | 0.35 | 5 | 0.6 | 4 | 2 | 0.6 |
| East and Central Asia | 36 | 3 | UMI | IPV/OPV | 6 | 0.25 | 60 | 0.35 | 5 | 0.9 | 5 | 1 | 0.6 |
| East and Central Asia | 36 | 4-10 | UMI | IPV/OPV | 6 | 0.25 | 60 | 0.35 | 5 | 0.98 | 5 | 1 | 0.6 |
| East and Central Asia | 37 | 1 | UMI | IPV/OPV | 7 | 0.2 | 120 | 0.35 | 5 | 0.9 | 4 | 2 | 0.6 |
| East and Central Asia | 37 | 2-10 | UMI | IPV/OPV | 7 | 0.2 | 120 | 0.35 | 5 | 0.98 | 5 | 2 | 0.6 |
| Europe | 38 | 1-2 | UMI | IPV/OPV | 6 | 0.4 | 180 | 0.45 | 6 | 0.6 | 3 | 3 | 0.8 |
| Europe | 38 | 3-5 | UMI | IPV/OPV | 6 | 0.4 | 180 | 0.45 | 6 | 0.9 | 4 | 2 | 0.8 |
| Europe | 38 | 6-10 | UMI | IPV/OPV | 6 | 0.4 | 180 | 0.45 | 6 | 0.98 | 5 | 1 | 0.8 |
| Europe | 39 | 1-2 | HIGH | IPV/OPV | 6 | 0.5 | 180 | 0.45 | 6 | 0.9 | 4 | 2 | 0.8 |
| Europe | 39 | 3-10 | HIGH | IPV/OPV | 6 | 0.5 | 180 | 0.45 | 6 | 0.98 | 5 | 1 | 0.8 |
| Europe | 40 | 1-2 | HIGH | IPV/OPV | 6 | 0.5 | 180 | 0.45 | 6 | 0.9 | 4 | 2 | 0.8 |
| Europe | 40 | 3-10 | HIGH | IPV/OPV | 6 | 0.5 | 180 | 0.45 | 6 | 0.98 | 5 | 1 | 0.8 |
| Europe | 41 | 1-4 | HIGH | IPV-only | 5 | 0.4 | 240 | 0.4 | 7 | 0.9 | 4 | 2 | 0.9 |
| Europe | 41 | 5-10 | HIGH | IPV-only | 5 | 0.4 | 240 | 0.4 | 7 | 0.98 | 5 | 1 | 0.9 |
| Europe | 42 | 1 | HIGH | IPV-only | 6 | 0.2 | 180 | 0.35 | 7 | 0.9 | 4 | 2 | 0.6 |
| Europe | 42 | 2 | HIGH | IPV-only | 5 | 0.2 | 180 | 0.35 | 7 | 0.9 | 4 | 2 | 0.8 |
| Europe | 42 | 3 | HIGH | IPV-only | 5 | 0.2 | 180 | 0.35 | 7 | 0.98 | 4 | 2 | 0.8 |
| Europe | 42 | 4-10 | HIGH | IPV-only | 5 | 0.2 | 180 | 0.35 | 7 | 0.98 | 5 | 1 | 0.8 |
| Europe | 43 | 1-2 | HIGH | IPV-only | 5 | 0.2 | 180 | 0.35 | 7 | 0.9 | 4 | 2 | 0.8 |
| Europe | 43 | 3 | HIGH | IPV-only | 5 | 0.2 | 180 | 0.35 | 7 | 0.98 | 4 | 2 | 0.8 |
| Europe | 43 | 4-9 | HIGH | IPV-only | 5 | 0.2 | 180 | 0.35 | 7 | 0.98 | 5 | 1 | 0.8 |
| Europe | 43 | 10 | HIGH | IPV-only | 6 | 0.2 | 180 | 0.35 | 7 | 0.98 | 5 | 1 | 0.6 |
| Europe | 44 | 1-5 | HIGH | IPV-only | 5 | 0.4 | 240 | 0.4 | 7 | 0.9 | 4 | 2 | 0.8 |
| Europe | 44 | 6-10 | HIGH | IPV-only | 5 | 0.4 | 240 | 0.4 | 7 | 0.98 | 5 | 1 | 0.8 |
| Europe | 45 | 1-6 | HIGH | IPV-only | 5 | 0.35 | 240 | 0.4 | 7 | 0.9 | 4 | 2 | 0.9 |
| Europe | 45 | 7-10 | HIGH | IPV-only | 5 | 0.35 | 240 | 0.4 | 7 | 0.98 | 5 | 1 | 0.9 |
| India | 46-47 | 1 | LMI | OPV-only | 13 | 0.2 | 180 | 0.35 | 1 | 0.3 | 2^a^ | 1 | 0.3 |
| India | 46-47 | 2-10 | LMI | OPV-only | 13 | 0.2 | 180 | 0.35 | 1 | 0.6 | 5 | 1 | 0.3 |
| India | 48 | 1-5 | LMI | OPV-only | 12 | 0.2 | 180 | 0.35 | 2 | 0.6 | 5 | 1 | 0.3 |
| India | 48 | 6-10 | LMI | OPV-only | 12 | 0.2 | 180 | 0.35 | 2 | 0.9 | 5 | 1 | 0.3 |
| India | 49-53 | 1-5 | LMI | OPV-only | 11 | 0.15 | 240 | 0.35 | 3 | 0.6 | 5 | 1 | 0.3 |
| India | 49-53 | 6-10 | LMI | OPV-only | 11 | 0.15 | 240 | 0.35 | 3 | 0.9 | 5 | 1 | 0.3 |
| India | 54-55 | 1-5 | LMI | OPV-only | 11 | 0.1 | 240 | 0.35 | 3 | 0.6 | 5 | 1 | 0.3 |
| India | 54-55 | 6-10 | LMI | OPV-only | 11 | 0.1 | 240 | 0.35 | 3 | 0.9 | 5 | 1 | 0.3 |
| India | 56-57 | 1-5 | LMI | OPV-only | 10 | 0.1 | 240 | 0.35 | 3 | 0.6 | 5 | 1 | 0.3 |
| India | 56-57 | 6-10 | LMI | OPV-only | 10 | 0.1 | 240 | 0.35 | 3 | 0.9 | 5 | 1 | 0.3 |
| Latin America & Caribbean | 58 | 1 | LMI | OPV-only | 8 | 0.05 | 180 | 0.3 | 3 | 0.6 | 3 | 3 | 0.3 |
| Latin America & Caribbean | 58 | 2-8 | LMI | OPV-only | 8 | 0.05 | 180 | 0.3 | 3 | 0.9 | 4 | 2 | 0.3 |
| Latin America & Caribbean | 58 | 9-10 | LMI | OPV-only | 8 | 0.05 | 180 | 0.3 | 3 | 0.98 | 5 | 1 | 0.3 |
| Latin America & Caribbean | 59 | 1 | UMI | OPV-only | 8 | 0.2 | 240 | 0.35 | 5 | 0.6 | 4 | 2 | 0.6 |
| Latin America & Caribbean | 59 | 2-6 | UMI | OPV-only | 8 | 0.2 | 240 | 0.35 | 5 | 0.9 | 5 | 2 | 0.6 |
| Latin America & Caribbean | 59 | 7-10 | UMI | OPV-only | 8 | 0.2 | 240 | 0.35 | 5 | 0.98 | 5 | 2 | 0.6 |
| Latin America & Caribbean | 60 | 1 | UMI | OPV-only | 7 | 0.1 | 0 | 0.35 | 5 | 0.6 | 4 | 2 | 0.6 |
| Latin America & Caribbean | 60 | 2-9 | UMI | OPV-only | 7 | 0.1 | 0 | 0.35 | 5 | 0.9 | 5 | 1 | 0.6 |
| Latin America & Caribbean | 60 | 10 | UMI | OPV-only | 7 | 0.1 | 0 | 0.35 | 5 | 0.98 | 5 | 1 | 0.6 |
| Latin America & Caribbean | 61 | 1-5 | UMI | IPV/OPV | 8 | 0.1 | 300 | 0.35 | 4 | 0.9 | 5 | 2 | 0.5 |
| Latin America & Caribbean | 61 | 6-10 | UMI | IPV/OPV | 8 | 0.1 | 300 | 0.35 | 4 | 0.98 | 5 | 1 | 0.5 |
| Latin America & Caribbean | 62 | 1 | UMI | IPV/OPV | 7 | 0.05 | 120 | 0.35 | 5 | 0.9 | 5 | 2 | 0.6 |
| Latin America & Caribbean | 62 | 2-5 | UMI | IPV/OPV | 7 | 0.05 | 120 | 0.35 | 5 | 0.98 | 5 | 2 | 0.6 |
| Latin America & Caribbean | 62 | 6-10 | UMI | IPV/OPV | 7 | 0.05 | 120 | 0.35 | 5 | 0.98 | 5 | 1 | 0.6 |
| Latin America & Caribbean | 63 | 1-5 | UMI | IPV/OPV | 8 | 0.05 | 300 | 0.35 | 4 | 0.9 | 5 | 2 | 0.5 |
| Latin America & Caribbean | 63 | 6-10 | UMI | IPV/OPV | 8 | 0.05 | 300 | 0.35 | 4 | 0.98 | 5 | 1 | 0.5 |
| North America | 64 | 1-4 | HIGH | IPV-only | 4 | 0.4 | 240 | 0.35 | 7 | 0.9 | 4 | 2 | 0.9 |
| North America | 64 | 5-10 | HIGH | IPV-only | 4 | 0.4 | 240 | 0.35 | 7 | 0.98 | 5 | 1 | 0.9 |
| North America | 65 | 1-4 | HIGH | IPV-only | 5 | 0.3 | 240 | 0.35 | 7 | 0.9 | 4 | 2 | 0.8 |
| North America | 65 | 5-10 | HIGH | IPV-only | 5 | 0.3 | 240 | 0.35 | 7 | 0.98 | 5 | 1 | 0.8 |
| North America | 66 | 1-4 | HIGH | IPV-only | 5 | 0.05 | 180 | 0.35 | 7 | 0.9 | 4 | 2 | 0.8 |
| North America | 66 | 5-9 | HIGH | IPV-only | 5 | 0.05 | 180 | 0.35 | 7 | 0.98 | 5 | 1 | 0.8 |
| North America | 66 | 10 | HIGH | IPV-only | 6 | 0.05 | 180 | 0.35 | 7 | 0.98 | 5 | 1 | 0.6 |
| South Asia | 67 | 1 | LOW | OPV-only | 12 | 0.3 | 180 | 0.3 | 1 | 0.9 | 4 | 2 | 0.3 |
| South Asia | 67 | 2-5 | LOW | OPV-only | 12 | 0.3 | 180 | 0.3 | 1 | 0.9 | 4 | 1 | 0.3 |
| South Asia | 67 | 6-10 | LOW | OPV-only | 12 | 0.3 | 180 | 0.3 | 1 | 0.98 | 5 | 1 | 0.3 |
| South Asia | 68 | 1 | LOW | OPV-only | 13 | 0.1 | 180 | 0.3 | 1 | 0.6 | 4 | 2 | 0.3 |
| South Asia | 68 | 2-7 | LOW | OPV-only | 13 | 0.1 | 180 | 0.3 | 1 | 0.9 | 4 | 1 | 0.3 |
| South Asia | 68 | 8-10 | LOW | OPV-only | 13 | 0.1 | 180 | 0.3 | 1 | 0.98 | 5 | 1 | 0.3 |
| South Asia | 69 | 1-3 | LMI | OPV-only | 8 | 0.15 | 180 | 0.4 | 4 | 0.9 | 4 | 2 | 0.3 |
| South Asia | 69 | 4-10 | LMI | OPV-only | 8 | 0.15 | 180 | 0.4 | 4 | 0.98 | 5 | 1 | 0.3 |
| South Asia | 70 | 1-10 | UMI | OPV-only | 7 | 0.1 | 240 | 0.35 | 5 | 0.98 | 5 | 1 | 0.6 |
| South Asia | 71 | 1-2 | UMI | IPV/OPV | 7 | 0.15 | 0 | 0.35 | 5 | 0.6 | 5 | 2 | 0.6 |
| South Asia | 71 | 3-5 | UMI | IPV/OPV | 7 | 0.15 | 0 | 0.35 | 5 | 0.9 | 5 | 1 | 0.6 |
| South Asia | 71 | 6-10 | UMI | IPV/OPV | 7 | 0.15 | 0 | 0.35 | 5 | 0.98 | 5 | 1 | 0.6 |

**Model input symbols:**[[5](#_ENREF_5), [12](#_ENREF_12)] R_0_, average annual basic reproduction number for WPV 1; α, seasonal amplitude of R_­0_, defined as the “proportional change in R_0_ due to seasonality” [[5, p. 717](#_ENREF_5)]; pd, peak day of R_0_; κ, strength of preferential mixing between age groups, defined as the “proportion of contacts reserved for individuals within the same mixing age group”[[5, p. 717](#_ENREF_5)]; trl, OPV take rate level (see Table A2); POL3, RI coverage with 3 or more non-birth doses; sil, SIA impact level (see Table A2) dt, detection threshold, defined as the cumulative incidence of paralytic polio cases per 10 million people required to detect an outbreak due to a poliovirus introduction in a subpopulation [[5](#_ENREF_5)], p^oro^, proportion of transmissions via oropharyngeal route

**Acronyms:** HIGH, high-income; IPV, inactivated poliovirus vaccine; LMI, lower middle-income; LOW, low-income; OPV, oral poliovirus vaccine; UMI, upper middle-income

**Footnote:** ^a^ Assumed to increase to SIA impact level 4 in 2010 as a result of intensification of efforts to interrupt wild poliovirus transmission in these reservoirs.

**Table A2: SIA impact and OPV take rate levels used in Table A1, generalized from situation-specific values used in prior work[**[**5**](#_ENREF_5)**,** [**12**](#_ENREF_12)**,** [**6**](#_ENREF_6)**,** [**7**](#_ENREF_7)**]**

| **Take rate level** | **Average per-dose take rate for:** | | | | | | | |
| --- | --- | --- | --- | --- | --- | --- | --- | --- |
|  | **tOPV1** | **tOPV2** | **tOPV3** | **mOPV1** | **mOPV2** | **mOPV3** | **bOPV1** | **bOPV3** |
| 1 | 0.35 | 0.60 | 0.27 | 0.45 | 0.60 | 0.45 | 0.42 | 0.42 |
| 2 | 0.40 | 0.65 | 0.32 | 0.52 | 0.65 | 0.52 | 0.50 | 0.50 |
| 3 | 0.45 | 0.70 | 0.35 | 0.60 | 0.70 | 0.60 | 0.54 | 0.54 |
| 4 | 0.50 | 0.72 | 0.40 | 0.70 | 0.65 | 0.65 | 0.60 | 0.60 |
| 5 | 0.55 | 0.73 | 0.45 | 0.80 | 0.85 | 0.75 | 0.70 | 0.70 |
| 6 | 0.60 | 0.74 | 0.50 | 0.85 | 0.90 | 0.80 | 0.75 | 0.75 |
| 7 | 0.65 | 0.75 | 0.55 | 0.90 | 0.95 | 0.85 | 0.80 | 0.80 |
| 8 | 0.70 | 0.80 | 0.60 | 0.95 | 0.98 | 0.90 | 0.85 | 0.85 |
| **SIA impact level** | **True coverage** | | | | **Repeated missed probability^a^** | | | |
| 1 | 0.15 | | | | 0.95 | | | |
| 2 | 0.35 | | | | 0.95 | | | |
| 3 | 0.50 | | | | 0.80 | | | |
| 4 | 0.80 | | | | 0.70 | | | |
| 5 | 0.95 | | | | 0.50 | | | |

**Acronyms:** bOPV(1,3), bivalent oral poliovirus vaccine (serotype 1 and serotype 3 component, respectively); mOPV(1,2.3), monovalent oral poliovirus vaccine (containing serotype 1, serotype 2, and serotype 3, respectively); SIA, supplemental immunization activity; tOPV(1,2,3), trivalent oral poliovirus vaccine (serotype 1, serotype 2, and serotype 3 component, respectively)

**Footnote:** ^a^ Defined as the proportion of targeted individuals missed by an SIA who were targeted and missed by the previous SIA[[6](#_ENREF_6)]

**Table A3: Assumptions for planned, preventive SIAs (pSIAs) in OPV-using blocks**

| **Time period** | **RI coverage (*POL3*)** | **SIA schedule showing: vaccine (day(s) of year)** |
| --- | --- | --- |
| **Before elimination of all WPVs in block^a^** | | |
| Year < – 3  (before bOPV introduction) | Any | tOPV (0, 30, 60, …, 360) |
| -3 ≤ year < 2  (before tOPV intensification) | 0.05 or 0.1 | tOPV (0, 240); bOPV (30, 60, 90, 120, 150, 180, 210, 270, 300, 330) |
|  | 0.3 | tOPV (0, 30); bOPV(60, 90,…,360) |
|  | 0.6 (R_0_ ≤ 10)  0.6 (R_0_ > 10) | tOPV (0); bOPV(30, 60, 90,…,360)  tOPV (0, 30); bOPV(60, 90,…,360) |
|  | 0.9 or 0.98 | tOPV (0, 30, 60,…,330) |
| 2 ≤ year < OPV2 cessation time  (after tOPV intensification) | 0.05 or 0.1 | tOPV (0, 30, 270, 300); bOPV (60, 90, 120, 150, 180, 210, 330) |
|  | 0.3 | tOPV (0, 30, 270); bOPV(60, 90, 120, 150, 180, 210, 300, 330) |
|  | 0.6 (R_0_ ≤ 10)  0.6 (R_0_ > 10) | tOPV (0); bOPV(30, 60, 90,…,360)  tOPV (0, 30, 270); bOPV(60, 90, 120, 150, 180, 210, 300, 330) |
|  | 0.9 or 0.98 | tOPV (0, 30, 60,…,330) |
| OPV2 cessation time ≤ year < OPV13 cessation time (after OPV2 cessation) | Any | bOPV (0, 30, 60, …, 360) |
| **After elimination of all WPVs in block** | | |
| Year < – 3  (before bOPV introduction) | 0.05 or 0.1 | tOPV (0, 40, 80, 140, 240, 300) |
|  | 0.3 | tOPV (0, 40, 80, 140, 240) |
|  | 0.6 (R_0_ ≤ 10)  0.6 (R_0_ > 10) | tOPV (0, 60, 120)  tOPV (0, 40, 80, 140, 240) |
|  | 0.9 | tOPV (0) |
|  | 0.98 (R_0_ ≤ 10)  0.98 (R_0_ > 10) | No SIAs  tOPV (0) |
| -3 ≤ year < 2  (before tOPV intensification) | 0.05 or 0.1 | tOPV (0, 40); bOPV (80, 140, 240, 300) |
|  | 0.3 | tOPV (0, 40); bOPV (80, 140, 240) |
|  | 0.6 (R_0_ ≤ 10)  0.6 (R_0_ > 10) | tOPV (0); bOPV (60, 120)  tOPV (0, 40); bOPV(80, 140, 240) |
|  | 0.9 | tOPV (0) |
|  | 0.98 (R_0_ ≤ 10)  0.98 (R_0_ > 10) | No SIAs  tOPV (0) |
| 2 ≤ year < OPV2 cessation time  (after tOPV intensification) | 0.05 or 0.1 | tOPV (0, 40, 80, 300); bOPV (140, 240) |
|  | 0.3 | tOPV (0, 40, 80); bOPV (140, 240) |
|  | 0.6 (R_0_ ≤ 10)  0.6 (R_0_ > 10) | tOPV (0, 60); bOPV (120)  tOPV (0, 40, 80); bOPV (140, 240) |
|  | 0.9 | tOPV (0) |
|  | 0.98 (R_0_ ≤ 10)  0.98 (R_0_ > 10) | No SIAs  tOPV (0) |
| OPV2 cessation time ≤ year < OPV13 cessation time (after OPV2 cessation) | 0.05 or 0.1 | bOPV (0, 40, 80, 140, 240, 300) |
|  | 0.3 | bOPV (0, 40, 80, 140, 240) |
|  | 0.6 (R_0_ ≤ 10)  0.6 (R_0_ > 10) | bOPV (0, 60, 120)  bOPV (0, 40, 80, 140, 240) |
|  | 0.9 | bOPV (0) |
|  | 0.98 (R_0_ ≤ 10)  0.98 (R_0_ > 10) | No SIAs  bOPV (0) |

**Acronyms:** bOPV, bivalent OPV; OPV, oral poliovirus vaccine; OPV13, serotype 1- and 3-containing OPV; OPV2, serotype-2-containing OPV; POL3, coverage with 3 or more non-birth RI doses; RI, routine immunization; SIA, supplemental immunization activity; tOPV, trivalent OPV; WPV, wild poliovirus

^a^ Annual frequency of SIAs starts at 2 and increase by one each year until all WPV eliminated from the block

**Table A4: Characteristics of stochastic iterations that led to OPV restart**

| **Year OPV restarts** | | | **Triggering event(s)** | | | | | | | | | | | |
| --- | --- | --- | --- | --- | --- | --- | --- | --- | --- | --- | --- | --- | --- | --- |
|  |  |  | **Time** | | **Type of event** | | **Sero-type** | **Block # (sub-pop #)** | | **Block with conditions like** | **Income level** | **Polio vaccine at T_0_** | **R0** | **POL3** |
| **OPV restarted with IPV5, IPV10 (n=2)** | | | | | | | | | | | | | | |
| 2051 | | | **2049.6** | Other release | | 3 | | 10 (5) | | W-Africa^a^ | LMI | OPV-only | 8 | 0.9 |
| 2043 | | | 2021.8  **2024.1** | iVDPV (CVID)  iVDPV (new) | | 1 | | 32 (0)  32 (9) | | N Pak/Afg | LOW | OPV-only  OPV-only | 11 | 0.1  0.6 |
| **OPV restarted with IPV through T_end_ (n=10)** | | | | | | | | | | | | | | |
| 2041 | | **2037.7** | | sIPV site release | | 1 | | 16 (3) | | India | LMI | OPV-only | 11 | 0.6 |
| 2051 | | **2049.6** | | Other release | | 3 | | 10 (5) | | W-Africa^a^ | LMI | OPV-only | 8 | 0.9 |
| 2040 | | **2037.1** | | sIPV site release | | 2 | | 6 (8) | | Egypt | LMI | OPV-only | 9 | 0.9 |
| 2046 | | **2043.5** | | sIPV site release | | 2 | | 54 (1) | | India | LMI | OPV-only | 11 | 0.6 |
| 2045 | | **2041.8** | | sIPV site release | | 1 | | 53 (1) | | India | LMI | OPV-only | 11 | 0.6 |
| 2052 | | **2049.9** | | sIPV site release | | 2 | | 57 (5) | | India | LMI | OPV-only | 10 | 0.9 |
| 2041 | | **2039.2** | | sIPV site release | | 2 | | 6 (5) | | Egypt | LMI | OPV-only | 9 | 0.9 |
| 2053 | | **2050.8** | | sIPV site release | | 2 | | 6 (8) | | Egypt | LMI | OPV-only | 9 | 0.9 |
| 2043 | | 2021.8  **2024.1** | | iVDPV (CVID)  iVDPV (new) | | 1 | | 32 (0)  32 (9) | | N Pak/Afg | LOW | OPV-only  OPV-only | 11 | 0.1  0.6 |
| 2047 | | **2043.2** | | sIPV site release | | 2 | | 50 (9) | | India | LMI | OPV-only | 11 | 0.9 |
| **OPV restarted with no IPV (n=6)** | | | | | | | | | | | | | | |
| 2051 | **2049.6** | | | | Other release | | 3 | | 10 (5) | W-Africa^a^ | LMI | OPV-only | 8 | 0.9 |
| 2030 | 2020.0  **2028.0** | | | | iVDPV (CVID)  iVDPV (new) | | 1 | | 33 (8) | Eurasia | LMI | OPV-only | 8 | 0.98 |
| 2044 | **2020.6** | | | | iVDPV (CVID) | | 2 | | 7 (8) | Africa-Arabia | LMI | OPV-only | 7 | 0.9 |
| 2036 | 2019.7 | | | | iVDPV (oPID  ) | | 1 | | 46 (2) | India | LMI | OPV-only | 13 | 0.6 |
| 2041 | 2021.4 | | | | iVDPV (CVID) | | 3 | | 3 (9) | E-Africa^b^ | LOW | OPV-only | 10 | 0.98 |
| 2034 | **2020.8** | | | | iVDPV (CVID) | | 2 | | 19 (8) | China | UMI | OPV-only | 6 | 0.98 |

**Acronyms (see Table 1 for policy abbreviations):** CVID, common variable immunodeficiency diseases; iVDPV, immunodeficiency-associated vaccine-derived poliovirus; LMI, lower middle-income; LOW, low-income; N Pak/Afg, North Pakistan and Afghanistan; noPID, other primary immunodeficiency disease (i.e., not CVID); OPV, oral poliovirus vaccine; R_0­_, average annual basic reproduction number for serotype 1 wild poliovrius, sIPV, inactivated poliovirus vaccine produced from Sabin seed strains; T_0_, start of analytical time horizon (i.e., first day of 2013); UMI, upper middle-income

Footnotes: ^a^ Not including Nigeria; ^b^e.g., Kenya

**Figure A1: Probability of effective introduction function (PEF)**


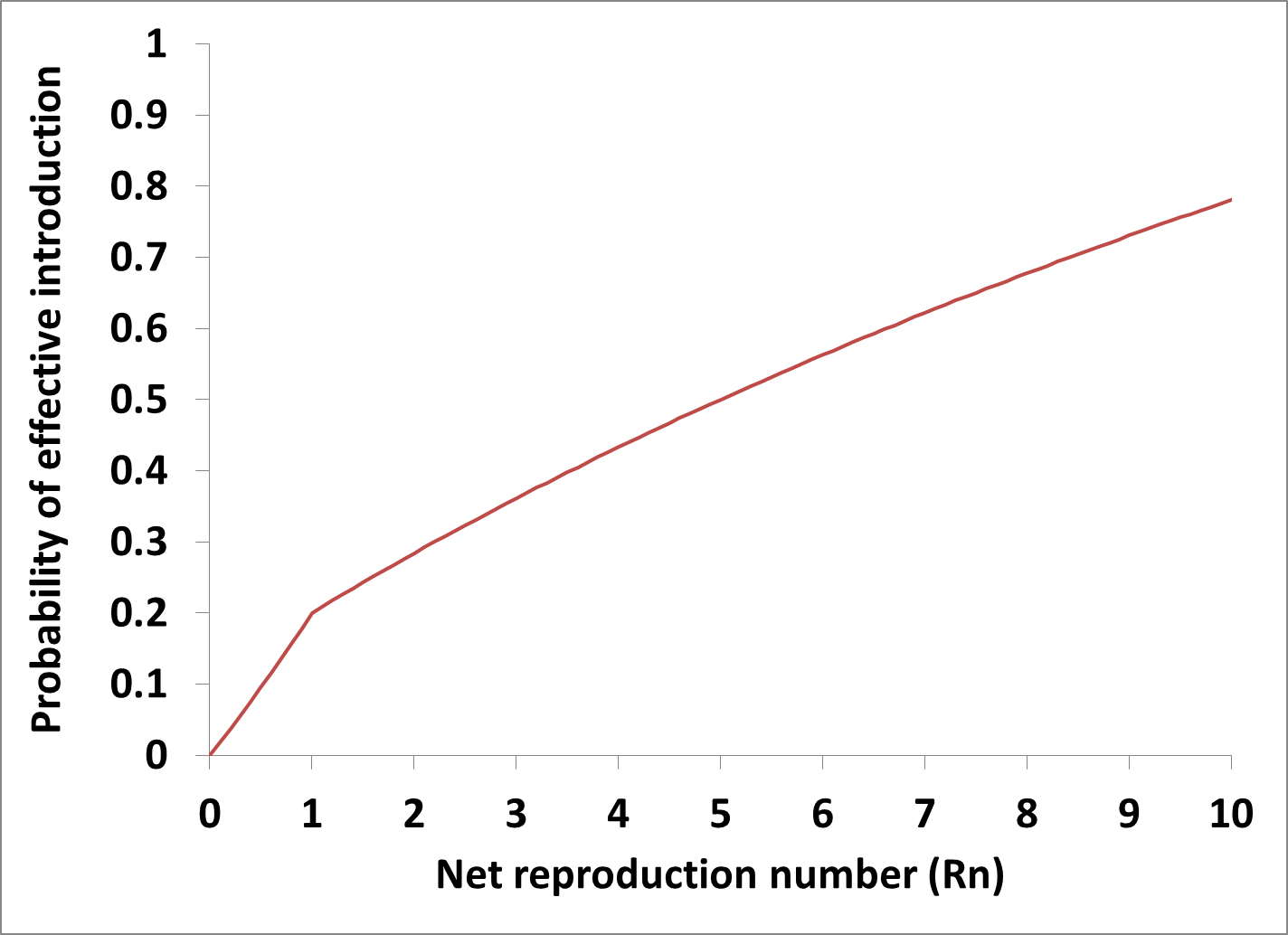


**Figure A2: Annual wild poliovirus (WPV) incidence during the 43-year burn-in period relative to the beginning of the analytic time horizon (T_0_, i.e., January 1, 2013) by serotype.**


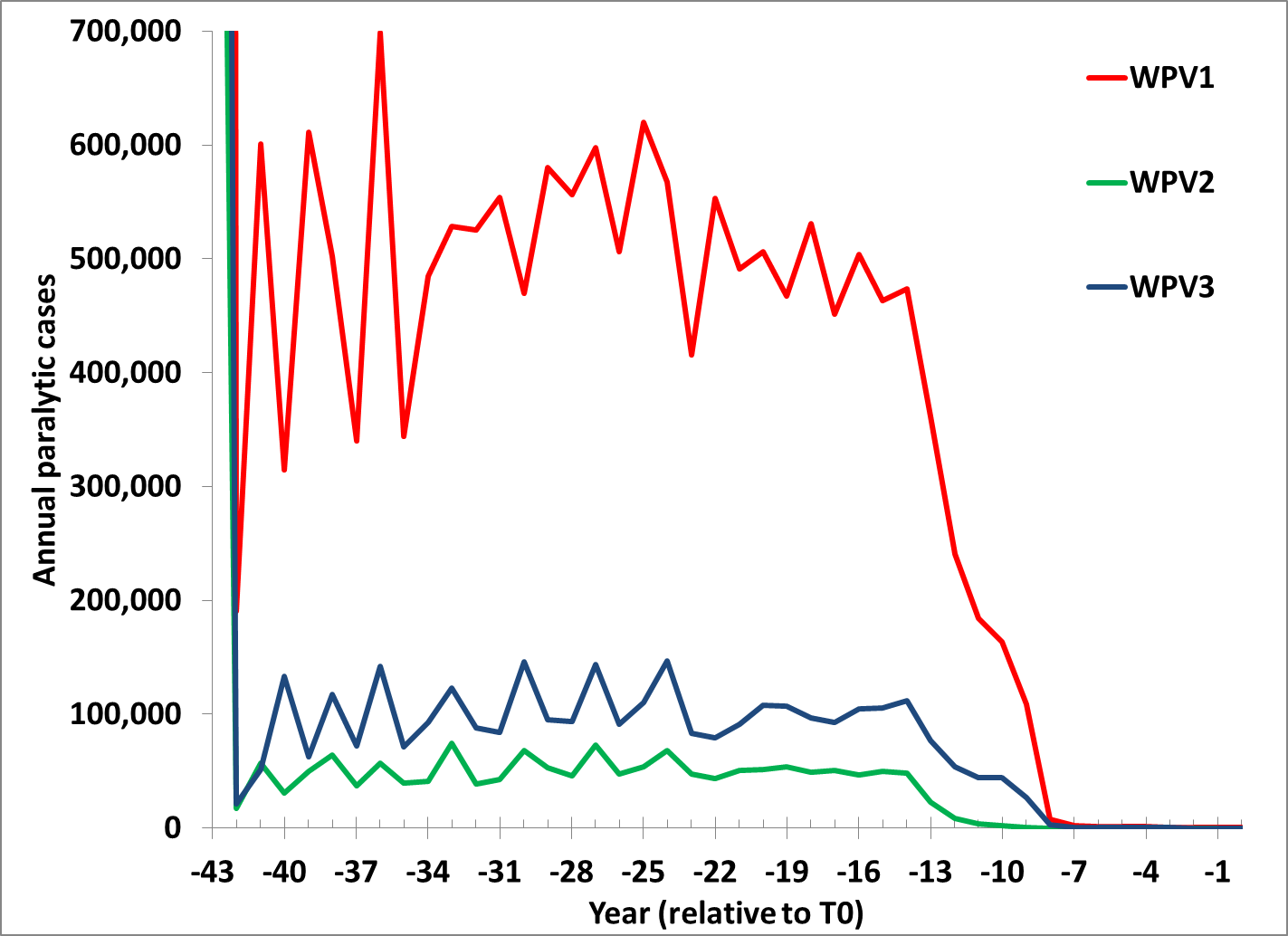


**REFERENCE**

1. Lopez AD, Mathers CD, Ezzati M, Jamison DT, Murray CJL. The Global Burden of Disease: A Comprehensive Assessment of Mortality and Disability from Diseases, Injuries, and Risk Factors in 1990 and Projected to 2020. Oxford, United Kingdom: Oxford University Press; 2006.

2. Duintjer Tebbens RJ, Pallansch MA, Chumakov KM, Halsey NA, Hovi T, Minor PD et al. Expert review on poliovirus immunity and transmission. Risk Anal. 2013;33(4):544-605.

3. Duintjer Tebbens RJ, Pallansch MA, Kim J-H, Burns CC, Kew OM, Oberste MS et al. Review: Oral Poliovirus Vaccine Evolution and Insights Relevant to Modeling the Risks of Circulating Vaccine-Derived Polioviruses (cVDPVs). Risk Anal. 2013;23(4):680-702.

4. Duintjer Tebbens RJ, Pallansch MA, Chumakov KM, Halsey NA, Hovi T, Minor PD et al. Review and assessment of poliovirus immunity and transmission: Synthesis of knowledge gaps and identification of research needs. Risk Anal. 2013;33(4):606-46.

5. Duintjer Tebbens RJ, Pallansch MA, Kalkowska DA, Wassilak SG, Cochi SL, Thompson KM. Characterizing poliovirus transmission and evolution: Insights from modeling experiences with wild and vaccine-related polioviruses. Risk Anal. 2013;23(4):703-49.

6. Duintjer Tebbens RJ, Kalkowska DA, Wassilak SGF, Pallansch MA, Cochi SL, Thompson KM. The potential impact of expanding target age groups for polio immunization campaigns. BMC Infect Dis. 2014;14(45). doi:10.1186/1471-2334-14-45.

7. Kalkowska DA, Duintjer Tebbens RJ, Grotto I, Shulman LM, Anis E, Wassilak SGF et al. Modeling options to manage type 1 wild poliovirus imported into Israel in 2013. J Infect Dis. 2015;211(11):1800-12.

8. Kalkowska DA, Duintjer Tebbens RJ, Thompson KM. Modeling strategies to increase population immunity and prevent poliovirus transmission in two high-risk areas in northern India. J Infect Dis. 2014;210(Suppl 1):S398-S411.

9. Kalkowska DA, Duintjer Tebbens RJ, Thompson KM. Modeling strategies to increase population immunity and prevent poliovirus transmission in the high-risk area of northwest Nigeria. J Infect Dis. 2014;210(Suppl 1):S412-S23.

10. Thompson KM, Pallansch MA, Duintjer Tebbens RJ, Wassilak SGF, Cochi SL. Modeling population immunity to support efforts to end the transmission of live polioviruses. Risk Anal. 2013;33(4):647-63.

11. Duintjer Tebbens RJ, Thompson KM. Modeling the potential role of inactivated poliovirus vaccine to manage the risks of oral poliovirus vaccine cessation. J Infect Dis. 2014;210(Suppl 1):S485-S97.

12. Thompson KM, Duintjer Tebbens RJ. Modeling the dynamics of oral poliovirus vaccine cessation. J Infect Dis. 2014;210(Suppl 1):S475-S84.

13. World Health Organization. WHO/UNICEF estimated coverage time series. WHO and UNICEF. 2012. <http://www.who.int/entity/immunization_monitoring/data/coverage_estimates_series.xls>. Accessed April 4 2012.

14. Duintjer Tebbens RJ, Pallansch MA, Thompson KM. Modeling the prevalence of immunodeficiency-associated long-term vaccine-derived poliovirus excretors and the potential benefits of antiviral drugs. BMC Infect Dis. 2015;In Press.
